# Efficacy and safety of Kangaroo mother care vs. conventional care during hospitalization for preterm and/or low birth weight infants: a meta-analysis with trial sequential analysis of randomized controlled trials

**DOI:** 10.3389/fmed.2025.1736973

**Published:** 2026-01-14

**Authors:** Li Li, Qiong Tan, Hongyu Li

**Affiliations:** Department of Gynecology and Obstetrics, The First Affiliated Hospital of Army Medical University, Chongqing, China

**Keywords:** conventional care, infants, Kangaroo mother care (KMC), low birth weight, mortality, preterm

## Abstract

**Background:**

Kangaroo mother care (KMC) plays a significant role in reducing mortality among preterm or low birth weight (LBW) infants. However, comprehensive evidence regarding various clinical outcomes during hospitalization for neonates receiving KMC remains limited. This meta-analysis focuses on hospital-based KMC and aims to evaluate its efficacy and safety for neonates during their hospital stay.

**Methods:**

A systematic search of the literature was undertaken across major databases, including PubMed, Web of Science, Embase, and the Cochrane Library, to identify randomized controlled trials (RCTs) published up to July 9, 2025. Binary outcomes were analyzed using risk ratio (RR), while continuous data were assessed using standardized mean difference (SMD) or mean difference (MD), each reported with corresponding 95% confidence intervals (CIs). To evaluate heterogeneity among studies, the Cochrane *Q* test, *I*^2^ statistic, and 95% prediction intervals (PIs) were utilized. Additionally, trial sequential analysis (TSA) was applied to mitigate potential type I and type II errors in the meta-analysis.

**Results:**

This meta-analysis synthesized data from 31 RCTs, encompassing 8561 preterm and/or LBW infants. The findings of this meta-analysis indicated that compared with conventional care, KMC significantly reduced in-hospital mortality [RR (95% CI) = 0.791 (0.696–0.899), 95% PI: 0.679–0.925] and 28-day all-cause mortality [RR (95% CI) = 0.810 (0.709–0.926), 95% PI: 0.605–1.085]. Furthermore, KMC was associated with shorter duration of hospitalization [MD (95% CI) = −0.809 (−1.601, −0.017), 95% PI: −3.219, 1.601] and notable improvements in growth parameters, including accelerated weight gain, increased length growth, and enhanced head circumference growth during the hospital stay (all *p* < 0.05). Regarding clinical safety, KMC was associated with lower risks of hypothermia, hyperthermia, apnea, sepsis, and necrotizing enterocolitis (all *p* < 0.05). Subgroup analyses further emphasized the marked clinical effectiveness and favorable safety profile of KMC, particularly in lower-middle-income countries.

**Conclusion:**

KMC is a clinically effective and safe intervention for the management of preterm and/or LBW neonates. Present evidence highlights its role in reducing neonatal mortality, expediting hospital discharge, fostering growth and developmental outcomes, as well as mitigating the incidence of complications. Moreover, the benefits of this intervention are particularly significant in lower-middle-income countries.

## 1 Introduction

Premature birth, defined as delivery before 37 weeks of pregnancy, and low birth weight (LBW), referring to infants weighing < 2,500 g, are significant contributors to neonatal and infant deaths (1). Globally, an estimated 13.4 million babies are delivered prematurely each year, and approximately one in four newborns are born frail and at risk, including those born preterm or small for gestational age (SGA) (2). These conditions not only represent the leading causes of mortality in children but also contribute substantially to long-term developmental and economic losses (2). The burden of preterm and low birth weight births is disproportionately high in low- and middle-income nations, where deficiencies in neonatal healthcare services exacerbate the problem (3).

Kangaroo mother care (KMC), which involves sustained skin-to-skin contact between the infant and the mother's chest alongside exclusive breastfeeding, is a highly effective intervention for reducing mortality among preterm and/or LBW neonates (4). The benefits of this approach have been well-documented, with evidence demonstrating its positive impact on key physiological parameters, including improved oxygenation, enhanced thermoregulation, stabilization of blood glucose levels, and overall homeostatic balance. Furthermore, KMC has been associated with advancements in neurodevelopmental and cognitive outcomes (5, 6). The close physical contact during KMC promotes exclusive breastfeeding and fosters a stronger maternal-infant bond (7). The implementation of KMC, however, varies widely across neonatal care practices, with its adoption being inconsistent across low-, middle-, and high-income countries. A Cochrane review found that providing KMC to stabilized LBW infants led to a 40% reduction in mortality compared to standard hospital care. This review also identified additional benefits, including lower rates of infection, improved weight gain, and increased prevalence of exclusive breastfeeding (8). Subsequent pooled analyses have reinforced these findings, demonstrating the substantial role of KMC in improving survival rates and reducing the risk of infections in preterm and/or LBW infants (9, 10).

Although systematic reviews have established the effectiveness of KMC, much of the existing evidence has been concentrated on its impact in reducing neonatal mortality and infection rates, with other dimensions of its clinical efficacy and safety remaining underexplored. Moreover, short-term outcomes observed during hospitalization may offer a more immediate and reliable measure for clinical benefits of KMC, as opposed to long-term follow-up results post-discharge, which are often influenced by a broader range of dynamic and unpredictable factors over time. Therefore, this study conducted a comprehensive analysis of randomized controlled trials (RCTs), with an emphasis on evaluating the efficacy and safety of hospital-based KMC compared with conventional neonatal care for LBW and/or preterm infants during their hospital stay. Further subgroup analyses were conducted to explore the clinical effects of KMC across countries with varying income levels. The findings aim to provide robust, evidence-based insights for policymakers and stakeholders to inform healthcare decisions and policies.

## 2 Methods

### 2.1 Study design

The meta-analysis was registered in the PROSPERO database under the identifier CRD420251146574. The research adhered to the principles and recommendations specified in the Preferred Reporting Items for Systematic Reviews and Meta-Analyses (PRISMA 2020) (11).

### 2.2 Search strategy

Two independent researchers conducted extensive literature searches across major international databases, including PubMed, Web of Science, Embase, and the Cochrane Library, to locate relevant studies published from inception through July 9, 2025. The search strategy targeted two primary domains: keywords associated with KMC (e.g., “kangaroo-mother care,” “kangaroo care,” “kangaroo mother method,” “skin to skin care,” “skin-skin care,” and “skin to skin contact”) and terms specific to infants, such as “preterm,” “premature,” “low birth weight,” “LBW,” “underweight,” and “VLBW”. The search was restricted to clinical RCTs. Detailed search methodologies were applied for each database, as outlined in Supplementary Files 1. The search was confined to studies involving human participants, with no restrictions on language. Furthermore, manual screening of reference lists from pertinent publications was performed to identify any additional eligible RCTs.

### 2.3 Inclusion and exclusion criteria

To be included, studies were required to fulfill the following criteria: (1) RCTs; (2) participants were preterm and/or LBW infants; (3) the intervention involved KMC, while the comparison group received conventional care; (4) outcomes assessed included measures of efficacy, such as in-hospital mortality, duration of hospitalization, exclusive breastfeeding at discharge, and growth parameters (e.g., weight, length, or head circumference gain during hospitalization). Safety outcomes encompassed adverse events (AEs) such as hypothermia, hyperthermia, apnea, sepsis, necrotizing enterocolitis (NEC), and hypoglycemia occurring during the hospital stay. Studies were excluded based on the following criteria: (1) non-RCT designs, including observational studies or single-arm trials; (2) study populations consisting of term or normal birth weight newborns; (3) interventions involving community-based KMC or outcomes unrelated to in-hospital endpoints; (4) animal research, case reports, letters, conference abstracts, or correspondences. To ensure the inclusion of more recent and clinically relevant evidence, studies published before 2000 were excluded from this meta-analysis.

### 2.4 Data extraction and quality assessment

Two reviewers independently extracted data from the included studies, utilizing a standardized data collection template. Extracted information included the name of the first author, publication year, country, study design, the caregiving interventions implemented in the intervention and control groups, sample size, birth weight, and gestational age in the intervention and control groups, stabilization status of the neonates and their use of invasive or non-invasive respiratory support. The methodological quality of the included RCTs was appraised using the modified Jadad scale (12), which evaluates critical elements such as the method of randomization, allocation concealment, blinding procedures, and documentation of participant withdrawals. Trials scoring between 0 and 3 were categorized as low quality, whereas those with scores of 4 or above were considered high quality. Discrepancies between reviewers were resolved through consensus discussions or, when necessary, by consulting a third investigator.

### 2.5 Statistical analysis

Binary and continuous outcomes were synthesized using risk ratio (RR), standardized mean difference (SMD), or mean difference (MD), each accompanied by their respective 95% confidence intervals (CIs). Heterogeneity among studies was evaluated using the Cochrane *Q* test, *I*^2^ statistic, and 95% prediction intervals (PIs), with significant heterogeneity defined as a *p*-value < 0.1 or an *I*^2^ value exceeding 50% (13, 14). A fixed-effects model was applied when heterogeneity was no significant, whereas a random-effects model, employing the DerSimonian and Laird approach, was used in the presence of substantial heterogeneity (15). Subgroup analyses were performed based on study quality or national income levels, categorized according to the World Bank classification (low-, lower-middle-, upper-middle-, and high-income countries) (16). Sensitivity analyses were conducted by systematically omitting individual studies to test the robustness of the combined estimates. Publication bias was assessed through visual inspection of funnel plots and further analyzed using Begg's and Egger's tests (17, 18). All statistical analyses were carried out using R software 4.3.2 and STATA 12.0. A two-tailed *p*-value of < 0.05 was considered statistically significant.

### 2.6 Trial sequential analysis

To mitigate the risk of type I and type II errors in the meta-analysis, trial sequential analysis (TSA) was employed (19). The analysis was performed using TSA software version 0.9.5.10 Beta, which calculated the required information size (RIS) and established sequential monitoring boundaries. When the cumulative Z-curve surpassed the RIS threshold or intersected the monitoring boundary, it indicated that the evidence was sufficient, and additional studies were unnecessary. The RIS was determined based on predefined parameters, including a two-sided significance level (α = 0.05), a statistical power of 80% (1 – β = 0.80), and an assumed 15% relative risk reduction (RRR).

## 3 Results

### 3.1 Study selection procedure

**Figure 1:**
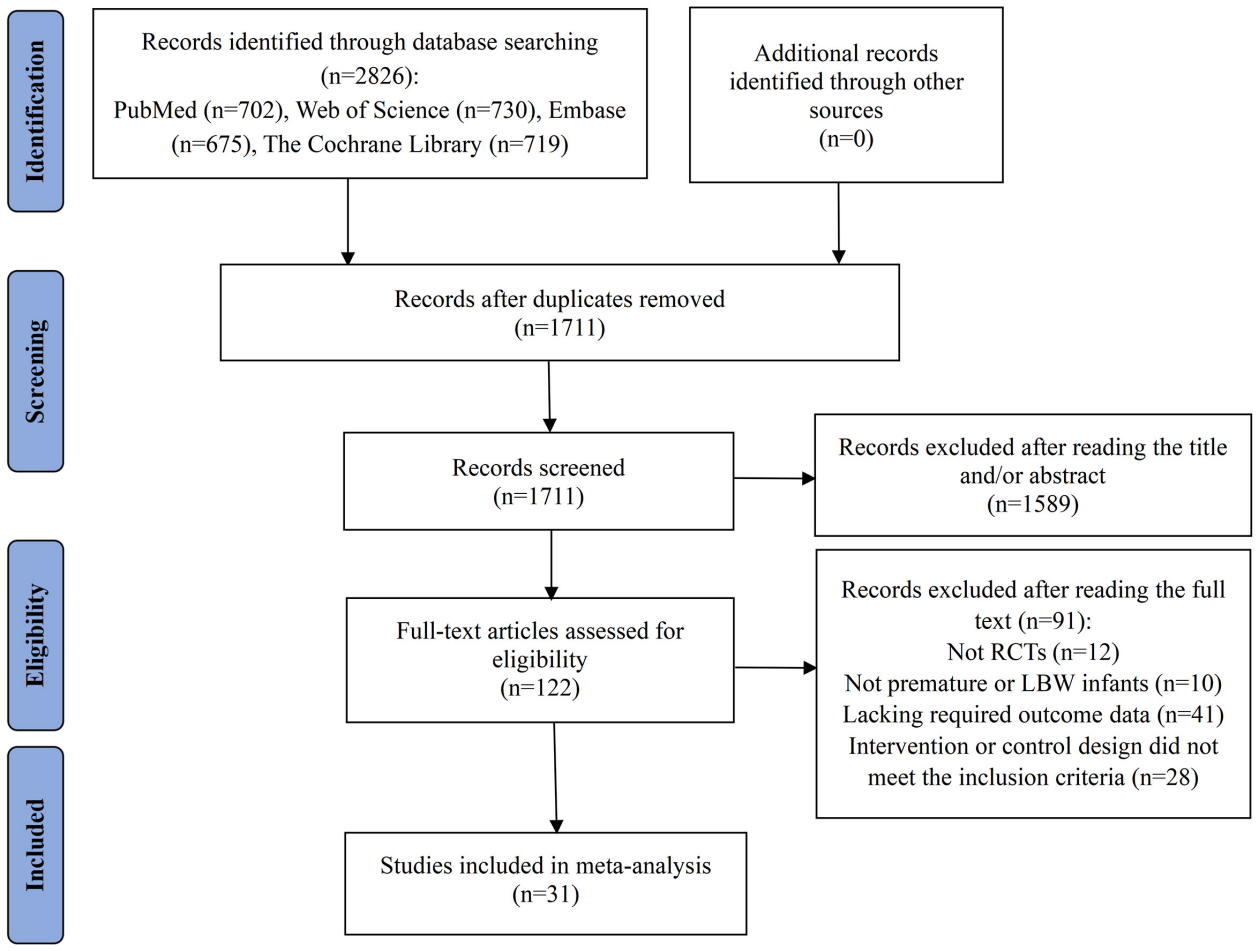
Flow diagram of the process of study selection. RCTs, randomized controlled trials; LBW, low birth weight.

The study selection process is summarized in Figure 1. An initial database search identified a total of 2,826 records. After duplicate entries were eliminated, 1,711 articles remained for title and abstract screening. Of these, 1,589 were excluded as they were unrelated to the research topic, leaving 122 articles for comprehensive full-text review. Upon further assessment, 91 studies were excluded for the following reasons: 12 were not RCTs, 10 did not involve preterm or LBW infants as the study population, 41 did not report the required outcome data, and 28 had intervention or control designs that did not meet the inclusion criteria. Ultimately, 31 studies fulfilled the eligibility requirements and were included in the meta-analysis (3, 20–49).

### 3.2 Study characteristics and quality assessment

The characteristics of the studies included in this meta-analysis are summarized in Table 1. To ensure the inclusion of more recent literature, studies published before 2000 were excluded. Consequently, 31 RCTs, all published in English between 2000 and 2025, were analyzed. These trials involved a total of 4,293 preterm and/or LBW infants receiving KMC, while 4,268 infants in the control groups underwent conventional care. Based on the World Bank classification, the studies were conducted in countries spanning various income levels: low-income nations (The Gambia, Ethiopia, and Uganda), lower-middle-income nations (Nepal, Egypt, India, Philippines, Bangladesh, Kenya, Vietnam, Ghana, Nigeria, Tanzania, and Pakistan), upper-middle-income nations (China, Indonesia, Malaysia, and Turkey), and high-income nations (United States, Norway, Australia, and Sweden). Quality assessment revealed that 18 studies were rated as high-quality, while 13 were categorized as low-quality, primarily due to challenges in blinding intervention procedures and outcome evaluations. Further details regarding the quality assessment process are provided in Supplementary Files 2.

**Table 1:** Characteristics of the included studies.

**Author (year)**	**Country**	**Design**	**Intervention description**	**Control description**	**Intervention group**			**Control group**			**Stabilisation status**	**Received invasive or non-invasive respiratory support**
					**Participants**	**Birth weight (g)**	**Gestational age (weeks)**	**Participants**	**Birth weight (g)**	**Gestational age (weeks)**		
WHO Immediate KMC Study Group et al. (2021)	Ghana, India, Malawi, Nigeria, and Tanzania	RCT	Immediate KMC	Conventional care in an incubator or a radiant warmer (including KMC after stabilization)	1609 infants with a birth weight between 1,000 and 1,799 g	1500 ± 200	32.6 ± 3.0	1602 infants with a birth weight between 1,000 and 1,799 g	1500 ± 200	32.6 ± 2.8	Unstable	Non-invasive
Brotherton et al. (2021)	The Gambia	RCT	Early continuous KMC before stabilization	Conventional care in an incubator or under a radiant heater (including continuous KMC after stabilization)	138 neonates with a birth weight < 2,000 g	Median (IQR): 1459 (1,204–1,650)	Median (IQR): 33 (31–34)	141 neonates with a birth weight < 2,000 g	Median (IQR): 1,436 (1,180–1,660)	Median (IQR): 32 (31–34)	Unstable	Non-invasive
Acharya et al. (2014)	Nepal	RCT	KMC	Conventional care (without KMC)	63 neonates with a birth weight < 2,000 g	1,385.87 ± 234.12	32.22 ± 2.4	63 neonates with a birth weight < 2,000 g	1,458.57 ± 172.66	32.54 ± 1.87	Stable	None
Worku et al. (2005)	Ethiopia	RCT	Early continuous KMC	Conventional care	62 infants with a birth weight < 2,000 g	Mean (Range): 1,514.8 (1,000–1,900)	32.42 (Mean)	61 infants with a birth weight < 2,000 g	Mean (Range): 1471.8 (930–1,900)	31.59 (Mean)	Unstable	Non-invasive
Ramadan et al. (2025)	Egypt	RCT	KMC	Conventional care	120 infants with a birth weight of < 2000 g	1,450.2 ± 135.3	32.1 ± 1.5	120 infants with a birth weight of < 2,000 g	1448.3 ± 137.6	32.0 ± 1.6	Stable	Non-invasive
Ali et al. (2009)	India	RCT	KMC	Conventional care (radiant warmers, cots in warm room)	58 neonates with a birth weight between 1200 and 1800 g	1607 ± 211	33.1 ± 2.3	56 neonates with a birth weight between 1,200 and 1,800 g	1615 ± 179	33.6 ± 2.29	Stable	NR
de Ocampo et al. (2021)	Philippines	RCT	KMC	Conventional care	26 neonates with a birth weight ≤ 1500 g	1,166.1 ± 0.19	32.5 ± 2.76	26 neonates with a birth weight ≤ 1,500 g	1,210.0 ± 0.23	32.1 ± 2.51	Stable	None
Nimbalkar et al. (2014)	India	RCT	Immediate KMC	Conventional care under radiant warmer	50 newborns weighing ≥ 1800 g	2,622.2 ± 398.69	37.8 ± 1.43	50 newborns weighing ≥ 1800 g	2,588.9 ± 443.25	37.7 ± 1.28	Stable	NR
Tumukunde et al. (2024)	Uganda	RCT	Early KMC	Conventional care in an incubator or radiant heater (with KMC possible after stabilisation)	1110 neonates with a birth weight between 700 and 2000 g	1,500.0 ± 300.0	32.3 ± 2.4	1111 neonates with a birth weight between 700 and 2,000 g	1,500.0 ± 300.0	32.3 ± 2.2	Unstable	Non-invasive
Gathwala et al. (2010)	India	RCT	KMC	Conventional care under a warmer or incubator (without KMC)	50 neonates with a birth weight of ≤ 1,800 g	1,690 ± 110	35.48 ± 1.20	50 neonates with a birth weight of ≤ 1,800 g	1,690 ± 120	35.04 ± 1.09	Stable	NR
Hoque et al. (2017)	Bangladesh	RCT	Intermittent KMC	Conventional care under radiant warmer and incubator	40 infants weighing 1,250 to 1,800 g	NR	NR	40 infants weighing 1,250 to 1,800 g	NR	NR	Stable	NR
Rojas et al. (2003)	USA	RCT	KMC	Conventional care (without KMC)	33 neonates with a birth weight of ≤ 1500 g and gestation ≤ 32 weeks	906 ± 245	26.6 ± 2.3	27 neonates with a birth weight of ≤ 1,500 g and gestation ≤ 32 weeks	939 ± 230	27.2 ± 2.3	Stable	Non-invasive
Ghavane et al. (2012)	India	RCT	KMC	Conventional care (incubator/ warmer)	71 infants with a birth weight < 1500 g	1,170 ± 191	30.8 ± 2.1	69 infants with a birth weight < 1,500 g	1,198 ± 194	30.7 ± 2.1	Stable	None
Lumbanraja (2016)	Indonesia	RCT	KMC	Conventional care in an incubator	20 infants with a birth weight of 1,000–2,500 g	1,882 ± 293.125	NR	20 infants with a birth weight of 1,000–2,500 g	1,803 ± 217.234	NR	Stable	None
Hake-Brooks et al. (2008)	USA	RCT	KMC	Conventional care	36 infants with a birth weight between 1300 and 3000 g	NR	32–36	30 infants with a birth weight between 1300 and 3000 g	NR	32–36	Stable	Non-invasive
Kristoffersen et al. (2023)	Norway	RCT	KMC	Conventional care	51 preterm infants born at gestational age 28–32 weeks with birth weight >1,000 g	1,436 ± 266	30.3 ± 1.1	57 preterm infants born at gestational age 28–32 weeks with birth weight >1,000 g	1,438 ± 257	30.3 ± 1.2	Stable	None
Boo et al. (2007)	Malaysia	RCT	KMC	Conventional care	64 infants with a birth weight < 1,501 g	1,515 ± 120	NR	62 infants with a birth weight < 1,501 g	1,492 ± 128	NR	Stable	Non-invasive
Kumbhojkar et al. (2016)	India	RCT	KMC	Conventional care	60 neonates with a birth weight of < 2,000 g	1677.16 ± 201.26	32.43 ± 1.8	60 neonates with a birth weight of < 2,000 g	1699 ± 199.34	32.40 ± 1.94	Stable	None
Chwo et al. (2002)	China	RCT	KMC	Conventional care (without KMC)	17 infants with gestation of 34–36 weeks	NR	34–36	17 infants with gestation of 34–36 weeks	NR	34–36	Stable	None
Singh et al. (2024)	India	RCT	KMC	Conventional care under radiant warmers	40 neonates with a birth weight of 1,000–1,800 g	NR	NR	40 neonates with a birth weight of 1,000–1,800 g	NR	NR	Stable	None
Pratiwi et al. (2009)	Indonesia	RCT	Early KMC	Conventional care	48 newborns with a birth weight of 1,500–2,250 g	2,033.75 ± 159.34	35.63 ± 2.27	45 newborns with a birth weight of 1,500–2,250 g	1,987.78 ± 176.06	35.53 ± 2.31	Stable	None
Mwendwa et al. (2012)	Kenya	RCT	KMC	Conventional care in incubators or cots	85 infants with a birth weight of 1,000–1,750 g	1,514 (Mean)	32.7 (Mean)	81 infants with a birth weight of 1,000–1,750 g	1,536 (Mean)	33.3 (Mean)	Stable	NR
Çaka et al. (2023)	Turkey	RCT	KMC	Conventional care	84 infants with a birth weight of 1,000–2,500 g and gestation 28–36 weeks	1,756 ± 503	32.10 ± 2.82	84 infants with a birth weight of 1,000–2,500 g and gestation 28–36 weeks	1,901 ± 557	32.78 ± 2.51	Stable	None
Kadam et al. (2005)	India	RCT	KMC	Conventional care	44 neonates with a birth weight of < 1,800 g	1,467 ± 228	33.3 ± 2.1	45 neonates with a birth weight of < 1,800 g	1,461 ± 217	34 ± 1.7	Stable	None
Chi Luong et al. (2016)	Vietnam	RCT	KMC	Conventional care (without KMC)	50 infants weighing between 1,500 and 2,490 g	2,060 ± 291.9	33.6 ± 1.8	50 infants weighing between 1,500 and 2,490 g	2,081 ± 259.3	33.9 ± 1.8	Unstable	Non-invasive and invasive
Ramanathan et al. (2001)	India	RCT	KMC	Conventional care under warmer or in the incubator	14 infants with a birth weight < 1,500 g	1,219 ± 186.4	Median (Range): 30.4 (28.8–34.1)	14 infants with a birth weight < 1,500 g	1,270.9 ± 170.4	Median (Range): 30.9 (29.0–33.3)	Stable	Non-invasive
Lode-Kolz et al. (2023)	Norway and Sweden	RCT	Immediate KMC	Conventional care (including KMC after the 6 first hours)	46 neonates with gestation of 28–33 weeks	Mean (Range): 1,572 (702–2,352)	Mean (Range): 31.3 (28.9–32.7)	45 neonates with gestation of 28–33 weeks	Mean (Range): 1,495 (555–2,440)	Mean (Range): 31.1 (28.6–32.9)	Unstable	Non-invasive and invasive
Roberts et al. (2000)	Australia	RCT	KMC	Conventional care	16 premature or SGA infants	1,562 ± 465	31.7 ± 3.1	14 premature or SGA infants	1,481 ± 409	31.2 ± 2.4	Stable	Non-invasive
Ricero-Luistro et al. (2021)	Philippines	RCT	KMC	Conventional care	35 neonates with a birth weight of 1,000–2,000 g and gestation < 36 weeks	1,559.43 ± 330.20	32.69 ± 2.60	35 neonates with a birth weight of 1,000–2,000 g and gestation < 36 weeks	1,551.29 ± 287.82	32.34 ± 2.22	Unstable	Non-invasive
Suman et al. (2008)	India	RCT	KMC	Conventional care	103 neonates with a birth weight of < 2,000 g	1,683.4 ± 235	35.3 ± 2.3	103 neonates with a birth weight of < 2,000 g	1,723.6 ± 242	35.9 ± 2.1	Stable	None
Manzoor et al. (2023)	Pakistan	RCT	KMC	Conventional care	50 infants with a birth weight of 1,500–2,499 g	NR	33.06 ± 2.132	50 infants with a birth weight of 1,500–2,499 g	NR	32.96 ± 2.03	Stable	None

RCT, randomized controlled trial; KMC, kangaroo mother care; IQR, interquartile range; NR, not reported; SGA, small for gestational age.

### 3.3 Overall and subgroup analysis of the efficacy outcomes

#### 3.3.1 In-hospital mortality and all-cause mortality at 28 days

8 studies examined the impact of KMC on in-hospital mortality among preterm and/or LBW infants. Given the absence of significant heterogeneity across the included trials, a fixed-effects model was applied for the statistical analysis (*I*^2^ = 0%, Tau^2^ = 0). The aggregated findings revealed a significant reduction in neonatal in-hospital mortality among infants receiving KMC compared to those who received conventional care [RR (95% CI) = 0.791 (0.696–0.899), 95% PI: 0.679–0.925] (Table 2, ). Subgroup analyses further demonstrated that the reduction in mortality was statistically significant in studies conducted in lower-middle-income countries or in trials assessed as high-quality (all *p* < 0.05). Conversely, studies performed in low-income settings or those rated as low-quality did not show a similar decrease in mortality rates (all *p* > 0.05) (Table 2, Supplementary Figure S1).

**Table 2:** Pooled effect and subgroup analysis of the efficacy outcomes of kangaroo mother care for preterm or low birth weight infants.

**Outcomes and subgroups**	**Number of studies**	**Meta-analysis**				**Heterogeneity**	
		**RR/MD/SMD**	**95% CI**	***p*** **value**	**95% PI**	*I* ^2^ **, Tau** ^2^	***p*** **value**
**In-hospital mortality**							
Overall	8	0.791	0.696–0.899	< 0.001	0.679–0.925	0%, 0	0.787
**World Bank classification**							
Low income	3	0.845	0.697–1.024	0.086	0.551–1.281	0%, 0	0.423
Lower-middle income	5	0.752	0.633–0.893	0.001	0.593–0.964	0%, 0	0.813
**Quality assessment**							
Low quality	3	0.627	0.383–1.026	0.063	0.212–1.823	0%, 0	0.936
High quality	5	0.804	0.704–0.917	0.001	0.668–0.973	0%, 0	0.594
All-cause mortality at 28 days	3	0.810	0.709–0.926	0.002	0.605–1.085	0%, 0	0.576
**Length of hospital stay**							
Overall	17	−0.809	−1.601, −0.017	0.045	−3.219, 1.601	99.9%, 1.1291	< 0.001
**World Bank classification**							
Low income	2	1.200	1.187, 1.213	< 0.001	1.114, 1.286	0%, 0	0.533
Lower-middle income	11	−1.490	−3.161, 0.181	0.081	−6.983, 4.003	87.8%, 5.3499	< 0.001
Upper-middle income	3	−0.854	−5.534, 3.827	0.721	−19.783, 18.076	83.9%, 13.6536	0.002
High income	1	2.000	−14.950, 18.950	0.817			
**Quality assessment**							
Low quality	5	−1.814	−4.910, 1.282	0.251	−10.261, 6.633	68.8%, 6.7612	0.012
High quality	12	−0.682	−1.562, 0.198	0.129	−3.221, 1.857	99.9%, 1.1288	< 0.001
**Fully breast-fed at hospital discharge**							
Overall	7	1.263	0.910–1.752	0.163	0.417–3.824	94.9%, 0.1772	< 0.001
**World Bank classification**							
Low income	1	1.000	0.964–1.038	0.985			
Lower-middle income	5	1.331	1.171–1.513	< 0.001	1.087–1.695	12.9%, 0.0025	0.332
High income	1	1.204	0.844–1.718	0.307			
**Quality assessment**							
Low quality	3	1.356	1.171–1.570	< 0.001	0.976–1.824	0%, 0	0.447
High quality	4	1.188	0.715–1.974	0.506	0.198–7.124	95.9%, 0.2496	< 0.001
**Weight gain rate**							
Overall	17	0.955	0.448, 1.461	< 0.001	−1.312, 3.221	97.2%, 1.0759	< 0.001
**World Bank classification**							
Low income	2	1.071	−1.408, 3.549	0.397	−26.723, 28.864	99.6%, 3.1858	< 0.001
Lower-middle income	10	1.151	0.762, 1.541	< 0.001	−0.240, 2.542	88.6%, 0.3386	< 0.001
Upper-middle income	3	0.620	−0.119, 1.358	0.100	−2.443, 3.683	89.2%, 0.3647	< 0.001
High income	2	0.254	−0.171, 0.678	0.242	−2.498, 3.005	0%, 0	0.390
**Quality assessment**							
Low quality	6	1.236	0.610, 1.862	< 0.001	−0.812, 3.285	89.0%, 0.5329	< 0.001
High quality	11	0.807	0.128, 1.487	0.020	−1.825, 3.440	98.1%, 1.2753	< 0.001
**Length gain rate**							
Overall	5	1.281	0.056, 2.505	0.041	−2.895, 5.456	97.1%, 1.8706	< 0.001
**World Bank classification**							
Lower-middle income	4	1.619	0.089, 3.149	0.038	−3.858, 7.096	97.8%, 2.3524	< 0.001
High income	1	0.000	−0.521, 0.521	0.999			
Quality assessment							
Low quality	1	5.756	4.853, 6.658	< 0.001			
High quality	4	0.269	0.066, 0.472	0.009	−0.060, 0.598	0%, 0	0.402
**Rate of head circumference gain**							
Overall	7	0.891	0.262, 1.520	0.006	−1.256, 3.038	93.9%, 0.6672	< 0.001
**World Bank classification**							
Lower-middle income	5	0.903	0.021, 1.786	0.045	−2.094, 3.900	95.8%, 0.9623	< 0.001
Upper-middle income	1	0.994	0.623, 1.365	< 0.001			
High income	1	0.761	0.220, 1.301	0.006			
**Quality assessment**							
Low quality	2	2.065	−0.463, 4.592	0.109	−26.133, 30.263	98.1%, 3.2624	< 0.001
High quality	5	0.457	0.038, 0.876	0.033	−0.865, 1.779	80.7%, 0.1809	< 0.001

**Figure 2:**
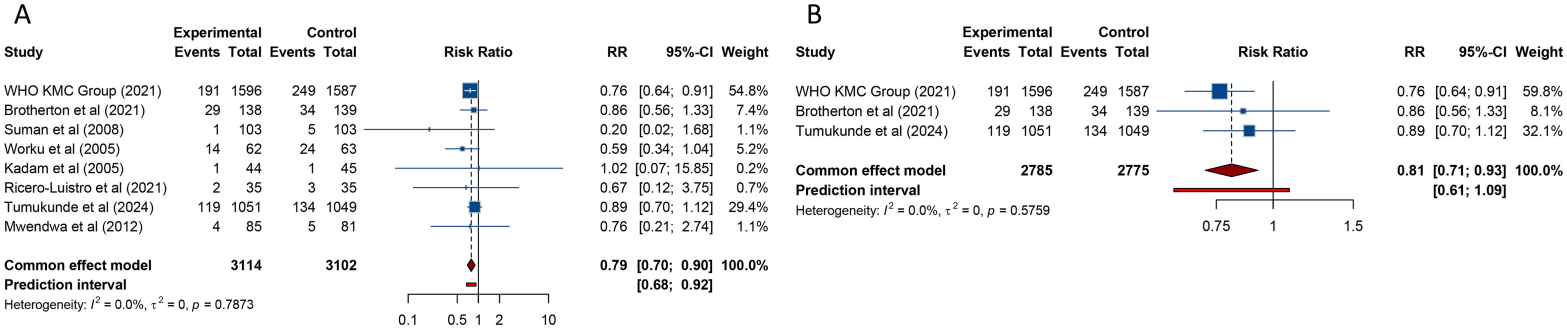
Forest plots of in-hospital mortality **(A)** and all-cause mortality at 28 days **(B)** after kangaroo mother care for preterm or low birth weight infants.

Of the 8 studies assessing in-hospital mortality, 3 high-impact publications specifically reported data on 28-day all-cause mortality. Pooled analysis of these studies showed that neonates receiving KMC experienced a significantly lower risk of death within 28 days compared to those receiving conventional care [RR (95% CI) = 0.810 (0.709–0.926), 95% PI: 0.605–1.085; *I*^2^ = 0%] (Table 2, Figure 2B). However, due to the limited number of studies available for this outcome, subgroup analysis was not performed.

#### 3.3.2 Length of hospital stay and fully breast-fed at hospital discharge

**Figure 3:**
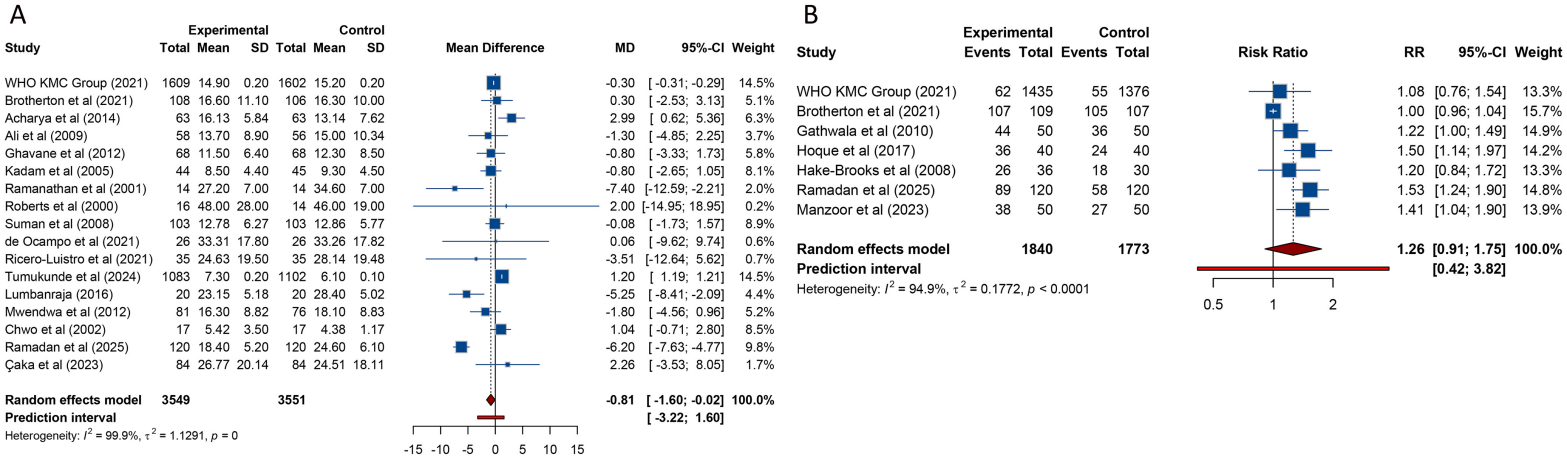
Forest plots of length of hospital stay **(A)** and fully breast-fed at hospital discharge **(B)** after kangaroo mother care for preterm or low birth weight infants.

17 studies investigated the influence of KMC on the duration of hospitalization among preterm and/or LBW infants. The meta-analysis revealed that neonates who received KMC experienced a significantly shorter hospital stay compared to those provided with conventional care [MD (95% CI) = −0.809 (−1.601, −0.017), 95% PI: −3.219, 1.601; *I*^2^ = 99.9%] (Table 2, Figure 3A). Notably, subgroup analysis showed that in low-income countries, infants receiving KMC had a longer hospital stay relative to those under conventional care [MD (95% CI) = 1.200 (1.187, 1.213), 95% PI: 1.114, 1.286; *I*^2^ = 0%]. In other subgroup analyses, no notable differences in hospitalization duration were identified between the two groups (all *p* > 0.05) (Table 2, Supplementary Figure S2).

Exclusive breastfeeding rates at discharge were examined in 7 studies comparing KMC with conventional care. The pooled analysis indicated no difference in exclusive breastfeeding rates between the two groups [RR (95% CI) = 1.263 (0.910–1.752), 95% PI: 0.417–3.824; *I*^2^ = 94.9%] (Table 2, Figure 3B). Subgroup analyses, however, revealed that in lower-middle-income countries or in studies classified as low quality, neonates receiving KMC had higher exclusive breastfeeding rates at discharge compared to those receiving conventional care (all *p* < 0.05). No significant differences were observed in other subgroups (all *p* > 0.05) (Table 2, Supplementary Figure S3).

#### 3.3.3 Rate of weight, length and head circumference gain

**Figure 4:**
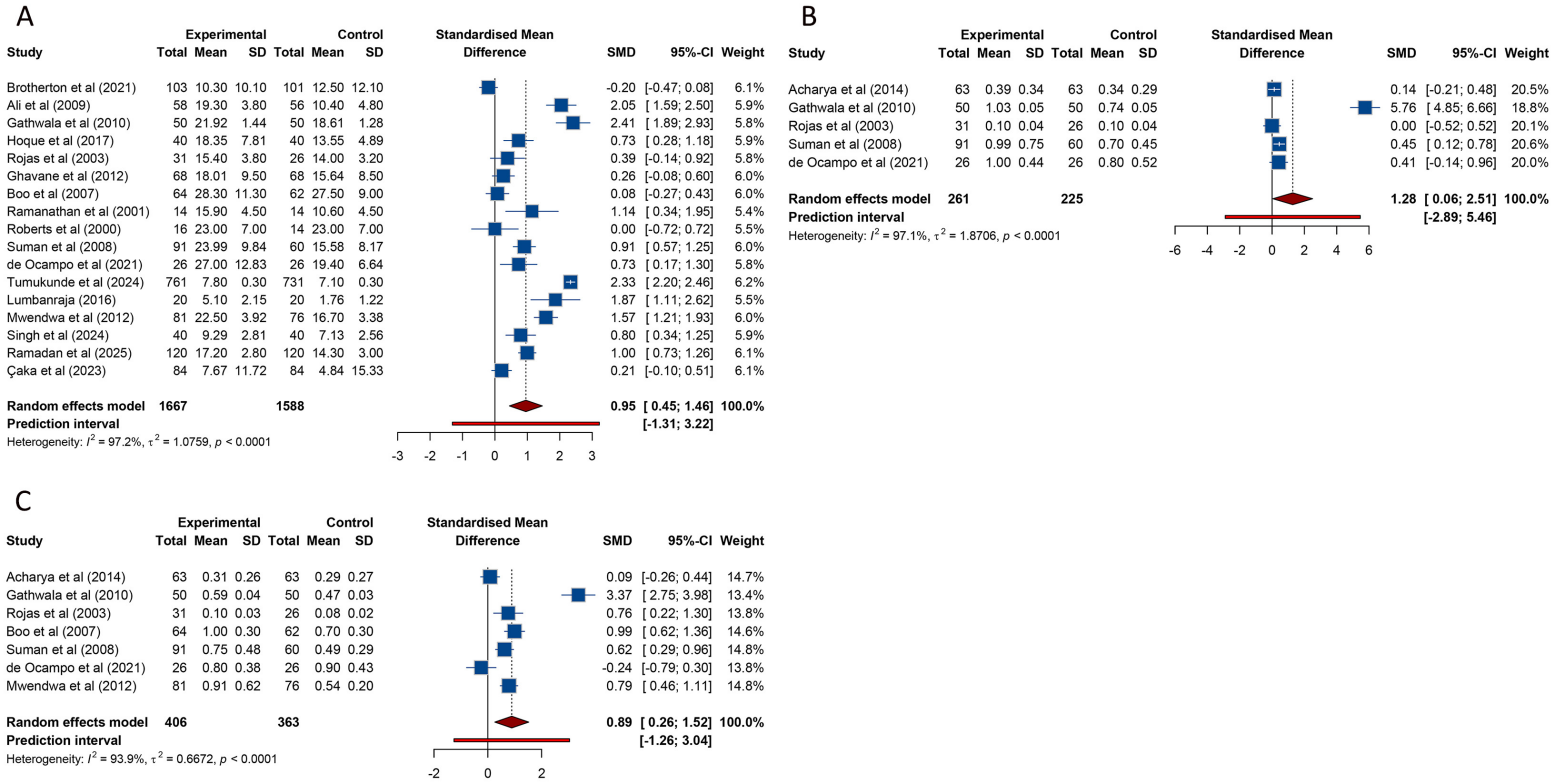
Forest plots of weight gain rate **(A)**, length gain rate **(B)** and rate of head circumference gain **(C)** after kangaroo mother care for preterm or low birth weight infants.

17 studies evaluated the impact of KMC on weight gain in hospitalized neonates. The pooled analysis demonstrated that neonates receiving KMC had a faster rate of weight gain compared with those receiving conventional care [SMD (95% CI) = 0.955 (0.448, 1.461), 95% PI: −1.312, 3.221; *I*^2^ = 97.2%] (Table 2, Figure 4A). This significant finding was validated only in lower-middle-income countries [SMD (95% CI) = 1.151 (0.762, 1.541), 95% PI: −0.240, 2.542; *I*^2^ = 88.6%], while no notable differences were identified in other income settings. Subgroup analyses stratified by study quality demonstrated that the accelerated weight gain in the KMC group was consistently observed in both high- and low-quality studies (all *p* < 0.05) (Table 2, Supplementary Figure S4).

The rate of length growth was assessed in 5 studies. The pooled findings showed that neonates receiving KMC experienced a more rapid increase in length compared to those receiving conventional care [SMD (95% CI) = 1.281 (0.056, 2.505), 95% PI: −2.895, 5.456; *I*^2^ = 97.1%] (Table 2, Figure 4B). This association was confirmed in lower-middle-income countries [SMD (95% CI) = 1.619 (0.089, 3.149), 95% PI: −3.858, 7.096; *I*^2^ = 97.8%]. Subgroup analyses based on study quality indicated that the faster rate of length gain in the KMC group was observed in high-quality studies [SMD (95% CI) = 0.269 (0.066, 0.472), 95% PI: −0.060, 0.598; *I*^2^ = 0%] (Table 2, Supplementary Figure S5).

7 studies reported on the rate of head circumference gain. The meta-analysis indicated that neonates receiving KMC had a faster rate of head circumference gain compared with those receiving conventional care [SMD (95% CI) = 0.891 (0.262, 1.520), 95% PI: −1.256, 3.038; *I*^2^ = 93.9%] (Table 2, Figure 4C). This significant result was validated in lower-middle-income countries [SMD (95% CI) = 0.903 (0.021, 1.786), 95% PI: −2.094, 3.900; *I*^2^ = 95.8%]. Subgroup analyses by study quality revealed that the accelerated head circumference growth associated with KMC was observed exclusively in high-quality studies [SMD (95% CI) = 0.457 (0.038, 0.876), 95% PI: −0.865, 1.779; I2 = 80.7%] (Table 2, Supplementary Figure S6).

### 3.4 Overall and subgroup analysis of the safety outcomes

#### 3.4.1 Hypothermia and hyperthermia

**Figure 5:**
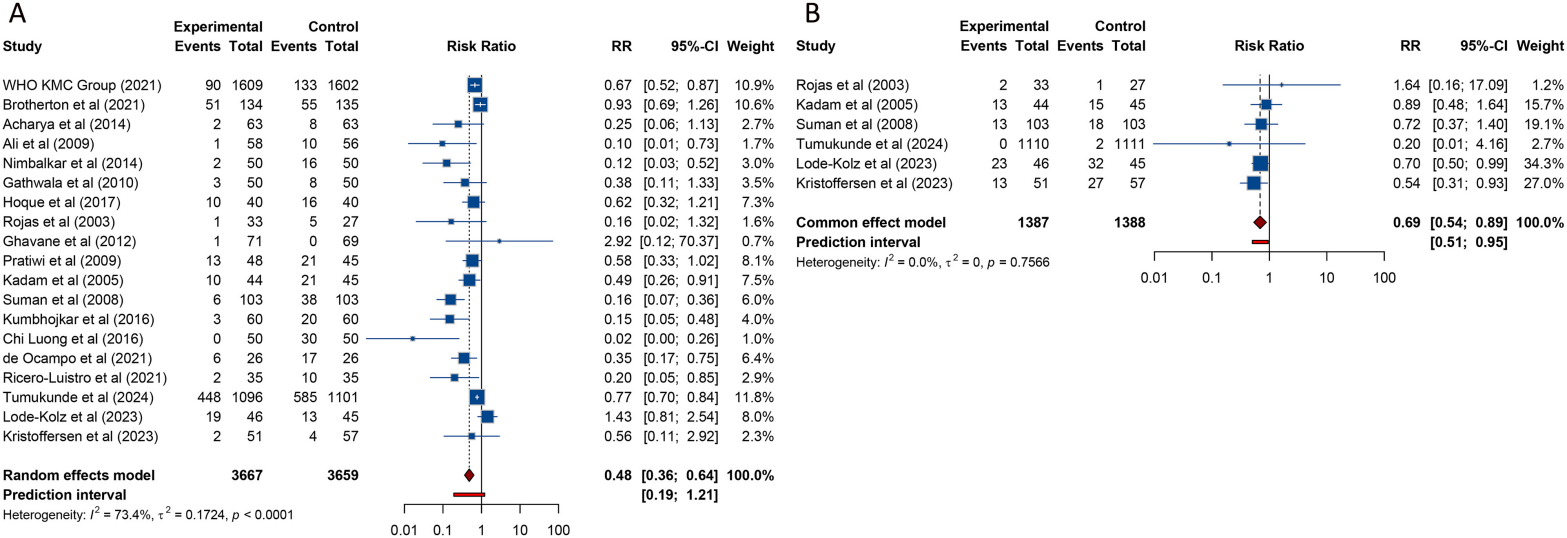
Forest plots of hypothermia **(A)** and hyperthermia **(B)** after kangaroo mother care for preterm or low birth weight infants.

19 studies investigated the incidence of hypothermia in both KMC and conventional care groups. The pooled analysis revealed that neonates receiving KMC had a significantly lower risk of hypothermia compared with those receiving conventional care [RR (95% CI) = 0.482 (0.363–0.638), 95% PI: 0.191–1.212; *I*^2^ = 73.4%] (Table 3, Figure 5A). This protective effect was consistently observed across low-income or lower-middle-income countries, as well as in both high-quality and low-quality studies (all *p* < 0.05) (Table 3, Supplementary Figure S7).

**Table 3:** Pooled effect and subgroup analysis of the safety outcomes of kangaroo mother care for preterm or low birth weight infants.

**Outcomes and subgroups**	**Number of studies**	**Meta-analysis**				**Heterogeneity**	
		**RR**	**95% CI**	***p*** **value**	**95% PI**	*I* ^2^ **, Tau** ^2^	***p*** **value**
**Hypothermia**							
Overall	19	0.482	0.363–0.638	< 0.001	0.191–1.212	73.4%, 0.1724	< 0.001
**World Bank classification**							
Low income	2	0.784	0.719–0.854	< 0.001	0.190–3.408	33.7%, 0.0064	0.220
Lower-middle income	13	0.303	0.191–0.483	< 0.001	0.073–1.263	67.1%, 0.3726	< 0.001
Upper-middle income	1	0.580	0.332–1.016	0.057			
High income	3	0.681	0.194–2.395	0.549	0.007–69.296	59.8%, 0.7425	0.083
**Quality assessment**							
Low quality	6	0.497	0.262–0.942	0.032	0.077–3.213	71.9%, 0.4210	0.003
High quality	13	0.456	0.325–0.642	< 0.001	0.175–1.194	75.5%, 0.1645	< 0.001
**Hyperthermia**							
Overall	6	0.689	0.535–0.886	0.004	0.506–0.954	0%, 0	0.757
**World Bank classification**							
Low income	1	0.200	0.010–4.165	0.299			
Lower-middle income	2	0.796	0.507–1.252	0.324	0.044–14.884	0%, 0	0.654
High income	3	0.649	0.482–0.875	0.005	0.351–1.244	0%, 0	0.532
**Quality assessment**							
Low quality	1	0.703	0.499–0.992	0.045			
High quality	5	0.681	0.485–0.956	0.027	0.423–1.111	0%, 0	0.624
**Apnea**							
Overall	11	0.547	0.415–0.721	< 0.001	0.156–1.823	47.4%, 0.2453	0.040
**World Bank classification**							
Low income	1	0.785	0.486–1.266	0.321			
Lower-middle income	9	0.418	0.294–0.594	< 0.001	0.108–1.759	45.0%, 0.2852	0.068
High income	1	3.273	0.388–27.580	0.276			
**Quality assessment**							
Low quality	2	0.371	0.084–1.637	0.191	–	79.4%, 0.9194	0.028
High quality	9	0.593	0.433–0.813	0.001	0.163–2.114	41.9%, 0.2328	0.088
**Sepsis**							
Overall	14	0.557	0.434–0.714	< 0.001	0.298–0.982	16.8%, 0.0528	0.270
**World Bank classification**							
Low income	1	0.972	0.611–1.547	0.906			
Lower-middle income	9	0.403	0.283–0.576	< 0.001	0.281–0.662	0%, 0	0.539
Upper-middle income	3	0.572	0.283–1.157	0.120	0.117–2.757	0%, 0	0.515
High income	1	0.511	0.189–1.383	0.186			
**Quality assessment**							
Low quality	4	0.373	0.199–0.698	0.002	0.076–2.172	21.1%, 0.1288	0.284
High quality	10	0.604	0.460–0.793	< 0.001	0.306–1.080	17.9%, 0.0495	0.278
**Necrotizing enterocolitis**							
Overall	5	0.575	0.358–0.923	0.022	0.185–1.819	17.3%, 0.0807	0.304
**World Bank classification**							
Low income	1	1.430	0.546–3.743	0.466			
Lower-middle income	3	0.410	0.227–0.739	0.003	0.112–1.494	0%, 0	0.979
High income	1	0.409	0.039–4.272	0.455			
**Quality assessment**							
Low quality	1	0.400	0.083–1.926	0.253			
High quality	4	0.598	0.364–0.982	0.042	0.099–3.897	35.2%, 0.1937	0.201
**Hypoglycemia**							
Overall	5	0.477	0.107–2.128	0.332	0.006–35.596	81.0%, 1.8303	< 0.001
**World Bank classification**							
Lower-middle income	4	0.320	0.061–1.664	0.175	0.002–57.091	84.7%, 1.9463	< 0.001
High income	1	5.577	0.274–113.504	0.264			
**Quality assessment**							
High quality	5	0.477	0.107–2.128	0.332	0.006–35.596	81.0%, 1.8303	< 0.001

The prevalence of hyperthermia was examined in 6 studies. The meta-analysis indicated that KMC was associated with a lower incidence of hyperthermia in neonates compared with conventional care [RR (95% CI) = 0.689 (0.535–0.886), 95% PI: 0.506–0.954; *I*^2^ = 0%] (Table 3, Figure 5B). Subgroup analyses further showed that this reduced incidence of hyperthermia with KMC was consistently observed in high-income countries as well as in high-quality or low-quality studies (all *p* < 0.05) (Table 3, Supplementary Figure S8).

#### 3.4.2 Apnea and sepsis

**Figure 6:**
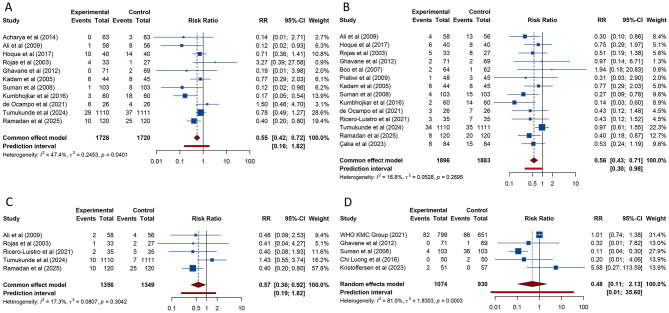
Forest plots of apnea **(A)**, sepsis **(B)**, necrotizing enterocolitis **(C)** and hypoglycemia **(D)** after kangaroo mother care for preterm or low birth weight infants.

11 studies examined the prevalence of apnea in neonates receiving KMC compared to those managed with conventional care. Meta-analysis results revealed that KMC was associated with a significantly reduced risk of apnea [RR (95% CI) = 0.547 (0.415–0.721), 95% PI: 0.156–1.823; *I*^2^ = 47.4%] (Table 3, Figure 6A). Subgroup analyses showed that this reduction was significant only in lower-middle-income countries [RR (95% CI) = 0.418 (0.294–0.594), 95% PI: 0.108–1.759; *I*^2^ = 45.0%] or in studies of high quality [RR (95% CI) = 0.593 (0.433–0.813), 95% PI: 0.163–2.114; *I*^2^ = 41.9%], while no significant findings were detected in other subgroups (all *p* > 0.05) (Table 3, Supplementary Figure S9).

The incidence of sepsis among hospitalized neonates was reported in 14 studies. The pooled analysis indicated that infants managed with KMC experienced a lower risk of sepsis compared to those receiving conventional care [RR (95% CI) = 0.557 (0.434–0.714), 95% PI: 0.298–0.982; *I*^2^ = 16.8%] (Table 3, Figure 6B). However, this significant association was confined to lower-middle-income countries [RR (95% CI) = 0.403 (0.283–0.576), 95% PI: 0.281–0.662; *I*^2^ = 0%], with no meaningful differences detected in other income classifications. Subgroup analyses stratified by study quality further confirmed that the reduction in sepsis incidence was consistently evident in both high-quality and low-quality studies (all *p* < 0.05) (Table 3, Supplementary Figure S10).

#### 3.4.3 NEC and hypoglycemia

5 studies assessed the prevalence of NEC in KMC and conventional care groups. The pooled results indicated that KMC was associated with a significantly decreased risk of NEC [RR (95% CI) = 0.575 (0.358–0.923), 95% PI: 0.185–1.819; *I*^2^ = 17.3%] (Table 3, Figure 6C). Subgroup analyses revealed that this protective effect was significant only in lower-middle-income countries [RR (95% CI) = 0.410 (0.227–0.739), 95% PI: 0.112–1.494; *I*^2^ = 0%] or in studies classified as high quality [RR (95% CI) = 0.598 (0.364–0.982), 95% PI: 0.099–3.897; *I*^2^ = 35.2%] (Table 3, Supplementary Figure S11).

Regarding neonatal hypoglycemia, 5 studies investigated its prevalence in KMC and conventional care groups. The meta-analysis found no evidence of a significant reduction in hypoglycemia rates among neonates receiving KMC compared to conventional care [RR (95% CI) = 0.477 (0.107–2.128), 95% PI: 0.006–35.596; *I*^2^ = 81.0%] (Table 3, Figure 6D). Subgroup analyses based on the World Bank classification or quality assessment also failed to identify any statistically significant differences (all *p* > 0.05) (Table 3, Supplementary Figure S12).

### 3.5 Trial sequential analysis results

**Figure 7:**
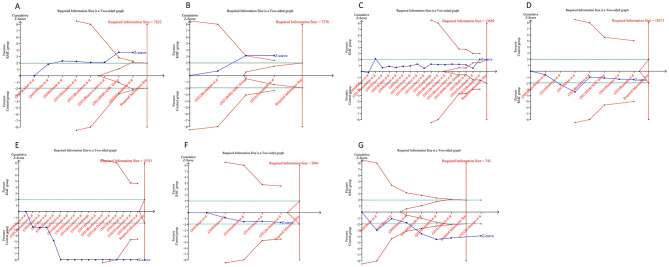
Trial sequential analysis of the efficacy outcomes after kangaroo mother care for preterm or low birth weight infants. **(A)** In-hospital mortality; **(B)** all-cause mortality at 28 days; **(C)** length of hospital stay; **(D)** fully breast-fed at hospital discharge; **(E)** weight gain rate; **(F)** length gain rate; **(G)** rate of head circumference gain. Uppermost and lowermost red curves represent trial sequential monitoring boundary lines for benefit and harm, respectively. Inner red lines represent the futility boundary. Blue line represents evolution of cumulative Z-score. Horizontal green lines represent the conventional boundaries for statistical significance. Cumulative Z-curve crossing the trial sequential monitoring boundary or the RIS boundary provides firm evidence of effect.

**Figure 8:**
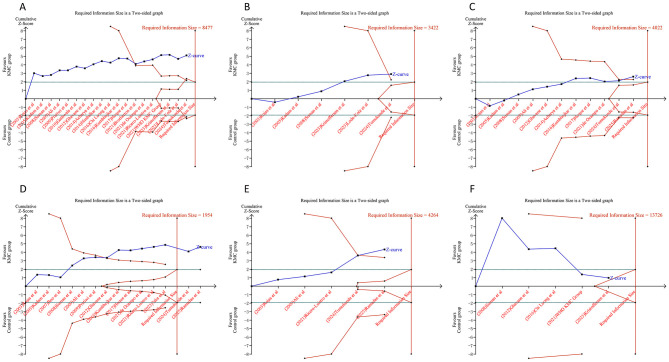
Trial sequential analysis of the safety outcomes after kangaroo mother care for preterm or low birth weight infants. **(A)** Hypothermia; **(B)** hyperthermia; **(C)** apnea; **(D)** sepsis; **(E)** necrotizing enterocolitis; **(F)** Hypoglycemia. Uppermost and lowermost red curves represent trial sequential monitoring boundary lines for benefit and harm, respectively. Inner red lines represent the futility boundary. Blue line represents evolution of cumulative Z-score. Horizontal green lines represent the conventional boundaries for statistical significance. Cumulative Z-curve crossing the trial sequential monitoring boundary or the RIS boundary provides firm evidence of effect.

Analysis of efficacy outcomes revealed that the cumulative Z-curves for in-hospital mortality, 28-day all-cause mortality, weight gain velocity, and head circumference growth surpassed the trial sequential monitoring or RIS thresholds. These findings indicated that the evidence supporting these endpoints is robust and conclusive, eliminating the need for additional trials. Conversely, the cumulative Z-curves for hospital stay duration, fully breast-fed at hospital discharge, and length gain rate did not meet the RIS and trial sequential monitoring boundaries, suggesting that the evidence for these measures remains uncertain and may be prone to false-positive results (Figure 7). Regarding safety outcomes, we observed that the cumulative Z-curves for all outcomes, except hypoglycemia, crossed either the RIS boundaries or the trial sequential monitoring boundaries (Figure 8).

### 3.6 Sensitivity analysis and publication bias

Sensitivity analyses and evaluations for publication bias were conducted for outcomes derived from at least 8 studies. To assess the impact of individual studies on the pooled results, a leave-one-out sensitivity analysis was conducted. For the pooled estimate of hospital stay duration, the proximity of the 95% CI to zero suggested that removing several individual studies could alter the statistical significance of the results. Additionally, sensitivity analyses for the remaining endpoints demonstrated relative robustness and stability (Supplementary Figure S13). Begg's and Egger's tests indicated potential publication bias among studies reporting the outcome of weight gain rate (Begg's test: *p* = 0.202; Egger's test: *p* = 0.046). However, subsequent analysis utilizing the trim-and-fill method showed no substantial changes in the direction and significance of the pooled results before and after adjustment, suggesting that the presence of publication bias did not substantially affect the conclusions. No evidence of significant publication bias was found for the remaining outcomes. Detailed funnel plots are presented in Supplementary Figure S14.

## 4 Discussion

The findings of this meta-analysis indicated that compared with conventional care, KMC significantly reduced in-hospital mortality and 28-day all-cause mortality among preterm and/or LBW infants. Furthermore, KMC was associated with shorter hospital stay and significantly improved growth parameters during hospitalization, including increased rates of weight gain, length growth, and head circumference growth. In terms of clinical safety, KMC was associated with lower risks of hypothermia, hyperthermia, apnea, sepsis, and NEC during hospitalization. However, no significant differences were identified between KMC and conventional care in the rates of exclusive breastfeeding at discharge and the incidence of neonatal hypoglycemia.

Our results align with earlier comprehensive reviews, highlighting that KMC plays a significant role in lowering neonatal death rates, shortening hospital stays, promoting faster growth, and mitigating the risk of various complications (8–10, 50, 51). Neonatal mortality remains the most clinically significant and patient-centered outcome in this context. The mechanisms underlying the benefits of KMC are multifaceted. By maintaining close physical proximity between mother and infant immediately after birth, the baby is more likely to be colonized by the mother's beneficial microbiota and to initiate breastfeeding earlier. Additionally, reduced handling of the infant by others helps lower the likelihood of infections (23, 30, 48). Continuous maternal observation, more frequent checks of the baby's glucose levels, and the elimination of stress (52) associated with separation from the mother may also contribute to the observed decrease in mortality rates. Research by Charpak et al. (53) suggests that the skin-to-skin positioning of KMC strengthens the maternal-infant bond, enhances the infant's reliance on the mother (54), and provides necessary stimulation to prevent apnea episodes (50). Simultaneously, this practice ensures sufficient warmth (55) to guard against deaths caused by hypothermia in preterm newborns (56).

Previous meta-analysis integrating relevant research have shown that KMC significantly shortens the duration of hospitalization when compared to standard care (51), a conclusion that aligns with the results of our study. Early discharge from neonatal units alleviates overcrowding, thereby decreasing the risk of hospital-acquired infections and lessening the financial burden on families. Moreover, it contributes to the reduction of overall healthcare costs (33, 57). Evidence suggested that KMC helps diminish pain responses during procedures such as heel-prick blood sampling in preterm infants, extends sleep duration, and supports growth and development, all of which contribute to a reduced hospital stay (58, 59). Among the key criteria for discharging preterm and/or LBW infants is adequate weight gain. Our study further confirmed that KMC promoted accelerated growth in weight, length, and head circumference, which may explain the observed reduction in hospital stay. Consistent with our results, prior studies have demonstrated that KMC not only reduces the time preterm infants spend in the hospital but also plays a pivotal role in fostering their overall growth and developmental progress (51, 60).

Hypothermia remains a well-recognized safety concern in preterm deliveries, whereas the risks associated with hyperthermia have been comparatively underexplored. In our analysis, the incidence of both hypothermia and hyperthermia was significantly reduced among neonates receiving KMC compared with those managed with conventional care, consistent with findings from previous studies involving infants born between 28^0^-32^6^ weeks of gestation (36). Supporting this, Mehler et al. (61) demonstrated that neonates exposed to 1 h of KMC in the delivery room had a higher mean skin temperature upon admission to the neonatal intensive care unit (NICU) (36.6 °C) compared to those receiving conventional care (36.1 °C). The physiological benefits of KMC are likely mediated through stabilization of cardiac and pulmonary function via the autonomic nervous system (62), coupled with enhanced microcirculation and improved tissue perfusion, resulting in better thermoregulation in LBW and/or preterm infants (63). Collectively, these findings provide robust evidence that early initiation of KMC is not only safe but also enhances thermoregulation in this vulnerable population.

A systematic review conducted in 2020 involving 416 preterm neonates identified a significant reduction in apneic episodes among infants receiving KMC (50). The findings of the present study further reinforce this evidence. Recent research has demonstrated enhanced cardiorespiratory stability in very preterm infants who underwent 6 h of KMC compared to those receiving conventional care immediately after birth (64). These results are consistent with earlier research suggesting that skin-to-skin contact contributes to improved respiratory function by supporting thermoregulation, attenuating stress responses, and fostering synchronized cardiorespiratory activity (65). Additionally, in line with the findings of Tas-Arslan et al. (66) this study suggested that KMC appears to be a safe intervention for neonates with respiratory compromise, potentially without increasing the likelihood of treatment failure.

A critical safety outcome associated with KMC is the significant reduction in hospital-acquired infections, including sepsis and NEC. These findings align with prior meta-analyses, which have documented a 65% decrease in nosocomial infections (67) and a 50% reduction in severe infections (68) among stable newborns managed with KMC. Such reductions in infection rates have been corroborated by multi-country studies, where improved survival free from infections was attributed to sustained maternal-infant contact, increased breastfeeding rates, and reduced exposure to hospital-related pathogens (22). While some critics have raised concerns about the potential limitations of KMC in settings with overcrowded facilities and inadequate nurse-to-patient ratios (69), the current multi-center trial, encompassing NICUs with varying levels of resources and capacity, consistently demonstrated infection prevention benefits across diverse healthcare environments (70). Furthermore, the degree to which KMC reduces infection risks may be influenced by specific nosocomial factors within the clinical setting. Risks such as exposure to contaminated fluids or inappropriate antibiotic use remain relevant regardless of the timing of KMC initiation. To optimize outcomes, infection control measures, such as rigorous hand hygiene practices among KMC providers and healthcare staff, should be prioritized and reinforced prior to implementing KMC programs (24).

Our subgroup analysis revealed that KMC significantly reduced in-hospital mortality among preterm and/or LBW infants in lower-middle-income countries. Additionally, KMC was associated with higher rates of exclusive breastfeeding at discharge, enhanced growth in weight, length, and head circumference, and a reduced incidence of hypothermia, apnea, sepsis, and NEC. These findings carry important implications for health policy, particularly in resource-limited settings. However, as the majority of studies included in this analysis were conducted in lower-middle-income countries, with limited representation from low-income, upper-middle-income, and high-income regions, the current evidence is insufficient to draw robust comparisons regarding the clinical efficacy and safety of KMC across diverse economic contexts. This underscores the need for future research to investigate these variations and provide a more comprehensive understanding of its impact in different income settings.

This study is subject to several limitations. First, data regarding race, ethnicity, and sex were not incorporated into the analysis due to inconsistent or absent reporting in the included studies. Second, the specific effects of immediate, early, or continuous KMC could not be adequately assessed, as the majority of included studies did not clearly distinguish between different types of KMC. Third, many RCTs analyzed had limited sample sizes, leading to small study effects and insufficient statistical power for certain outcomes, with some trials reporting no events in either group. Fourth, a lack of standardized definitions for KMC and inconsistent implementation across intervention and control groups further contributed to heterogeneity in the meta-analysis, potentially underestimating the true impact of KMC. Standardized reporting of key definitions and practices in future RCTs is essential to enhance comparability across studies. Fifth, the inability to achieve complete blinding of caregivers, given the inherently visible nature of skin-to-skin contact, likely introduced performance bias. This methodological limitation may have influenced the overall quality of some studies included in the analysis.

## 5 Conclusion

In conclusion, our findings indicated that KMC is beneficial for preterm and/or LBW infants, as evidenced by reductions in neonatal in-hospital mortality and 28-day all-cause mortality, shorter hospital stays, and accelerated growth in weight, length, and head circumference. Additionally, KMC was associated with a decreased risk of AEs, including hypothermia, hyperthermia, apnea, sepsis, and NEC. Subgroup analyses further highlighted the substantial clinical effectiveness and favorable safety profile of KMC, particularly in resource-limited settings within lower-middle-income countries. Future research should focus on evaluating the efficacy and safety of KMC in clinical practice across countries with varying income levels.

## Data Availability

The original contributions presented in the study are included in the article/Supplementary material, further inquiries can be directed to the corresponding author.
