# Supplementary material for: Efficacy and safety of Kangaroo mother care vs. conventional care during hospitalization for preterm and/or low birth weight infants: a meta-analysis with trial sequential analysis of randomized controlled trials
Source: Front Med (Lausanne). 2026 Jan 14;12:1736973. doi: 10.3389/fmed.2025.1736973 (PMC12847293; doi:10.3389/fmed.2025.1736973)
Supplement: Supplementary file 1 [file Data_Sheet_1.docx]

**Web of Science 730**

#1 TS=((kangaroo-mother care) OR (kangaroo mother care) OR (kangaroo care) OR (kangaroo mother method) OR (skin to skin care) OR (skin to skin contact) OR (skin-skin care) OR (skin-to-skin contact))

#2 TS=((preterm) OR (premature) OR (low birth weight) OR (LBW) OR (underweight) OR (VLBW))

#3 TS=((randomized controlled trial) OR (controlled clinical trial) OR (randomized) OR (clinical trial))

#4 #1 AND #2 AND #3

**PubMed 702**

#1 (kangaroo-mother care) OR (kangaroo mother care) OR (kangaroo care) OR (kangaroo mother method) OR (skin to skin care) OR (skin to skin contact) OR (skin-skin care) OR (skin-to-skin contact)

#2 (preterm) OR (premature) OR (low birth weight) OR (LBW) OR (underweight) OR (VLBW)

#3 (randomized controlled trial) OR (controlled clinical trial) OR (randomized) OR (clinical trial)

#4 #1 AND #2 AND #3

**The Cochrane Library 719**

#1 ((kangaroo-mother care) OR (kangaroo mother care) OR (kangaroo care) OR (kangaroo mother method) OR (skin to skin care) OR (skin to skin contact) OR (skin-skin care) OR (skin-to-skin contact)):ti,ab,kw

#2 ((preterm) OR (premature) OR (low birth weight) OR (LBW) OR (underweight) OR (VLBW)):ti,ab,kw

#3 ((randomized controlled trial) OR (controlled clinical trial) OR (randomized) OR (clinical trial)):ti,ab,kw

#4 #1 AND #2 AND #3

**Embase 675**

#1 'kangaroo-mother care'/exp OR 'kangaroo mother care'/exp OR 'kangaroo care'/exp OR 'kangaroo mother method'/exp OR 'skin to skin care'/exp OR 'skin to skin contact'/exp OR 'skin-skin care' OR 'skin-to-skin contact'

#2 preterm OR 'premature'/exp OR 'low birth weight'/exp OR lbw OR 'underweight'/exp OR 'vlbw'/exp

#3 'randomized controlled trial'/exp OR 'controlled clinical trial'/exp OR randomized OR 'clinical trial'/exp

#4 #1 AND #2 AND #3
