# Supplementary material for: Efficacy and safety of Kangaroo mother care vs. conventional care during hospitalization for preterm and/or low birth weight infants: a meta-analysis with trial sequential analysis of randomized controlled trials
Source: Front Med (Lausanne). 2026 Jan 14;12:1736973. doi: 10.3389/fmed.2025.1736973 (PMC12847293; doi:10.3389/fmed.2025.1736973)

**FIGURE S1** Subgroup analysis of in-hospital mortality. (A) Subgroup = Low income; (B) Subgroup = Lower-middle income; (C) Subgroup = Low quality; (D) Subgroup = High quality.


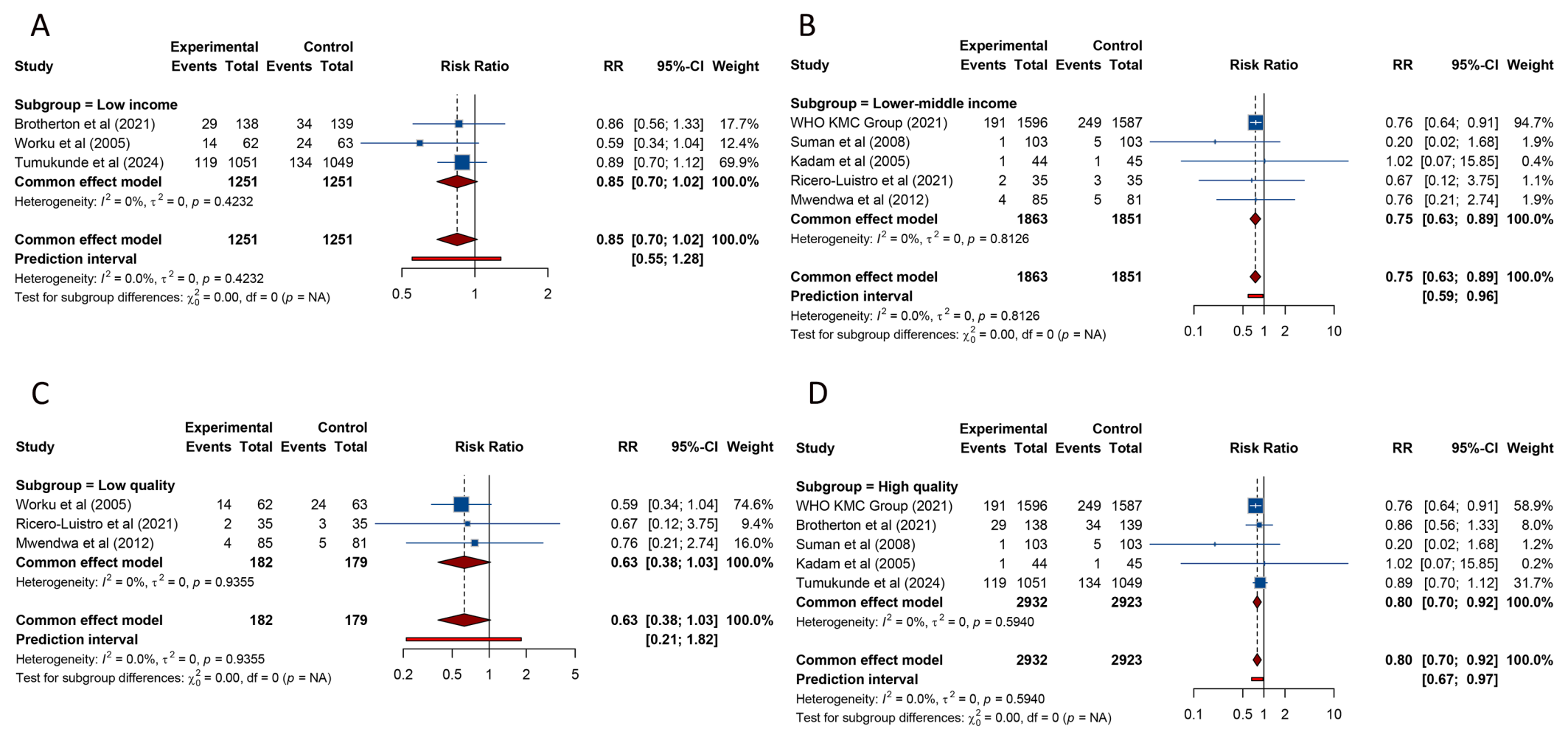


**FIGURE S2** Subgroup analysis for length of hospital stay. (A) Subgroup = Low income; (B) Subgroup = Lower-middle income; (C) Subgroup = Upper-middle income; (D) Subgroup = High income; (E) Subgroup = Low quality; (F) Subgroup = High quality.


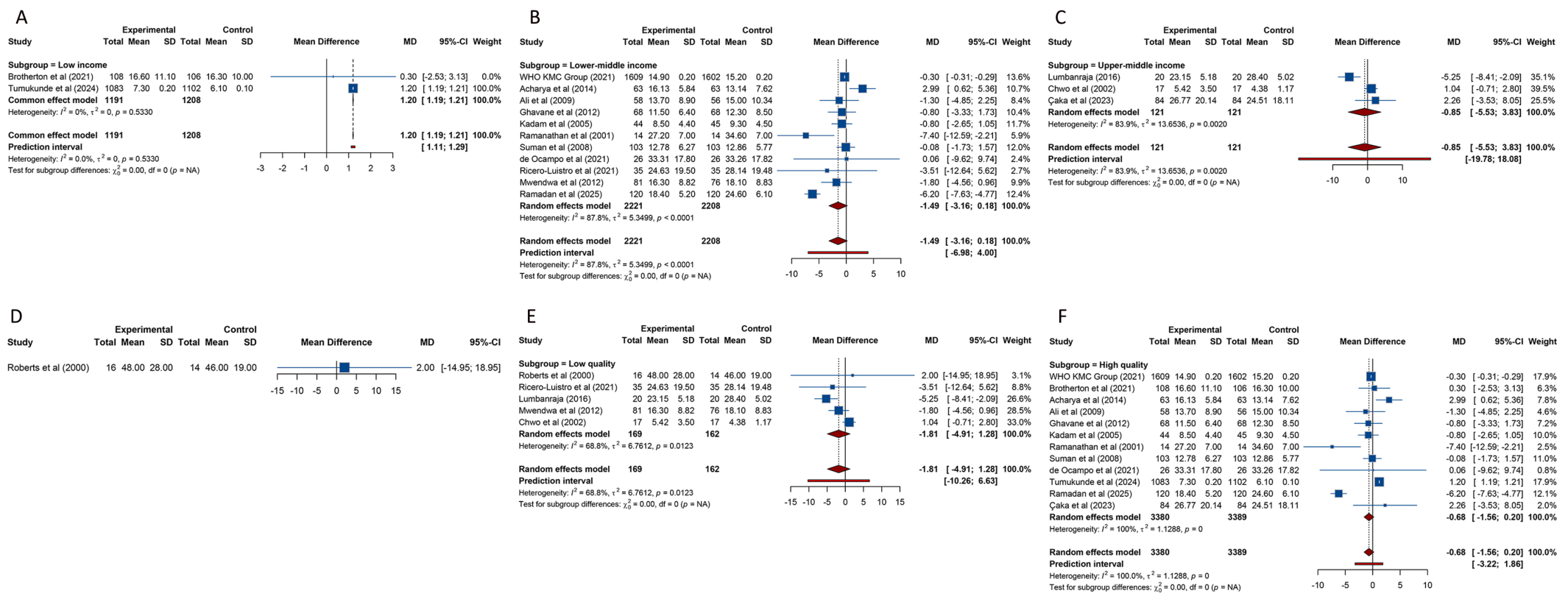


**FIGURE S3** Subgroup analysis of fully breast-fed at hospital discharge. (A) Subgroup = Low income; (B) Subgroup = Lower-middle income; (C) Subgroup = High income; (D) Subgroup = Low quality; (E) Subgroup = High quality.


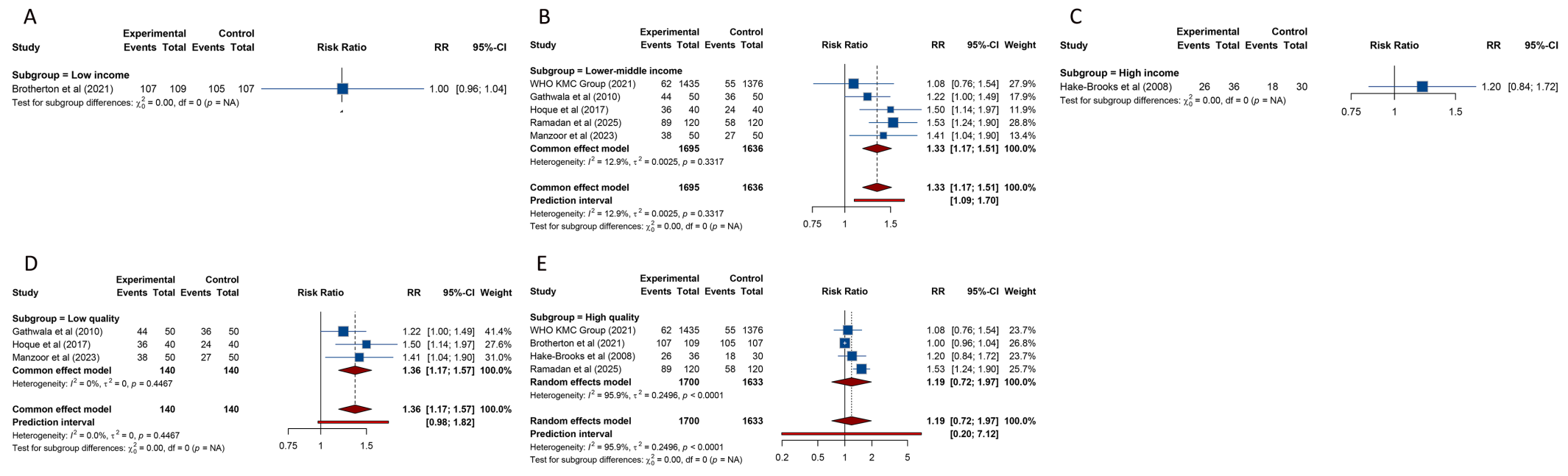


**FIGURE S4** Subgroup analysis of weight gain rate. (A) Subgroup = Low income; (B) Subgroup = Lower-middle income; (C) Subgroup = Upper-middle income; (D) Subgroup = High income; (E) Subgroup = Low quality; (F) Subgroup = High quality.


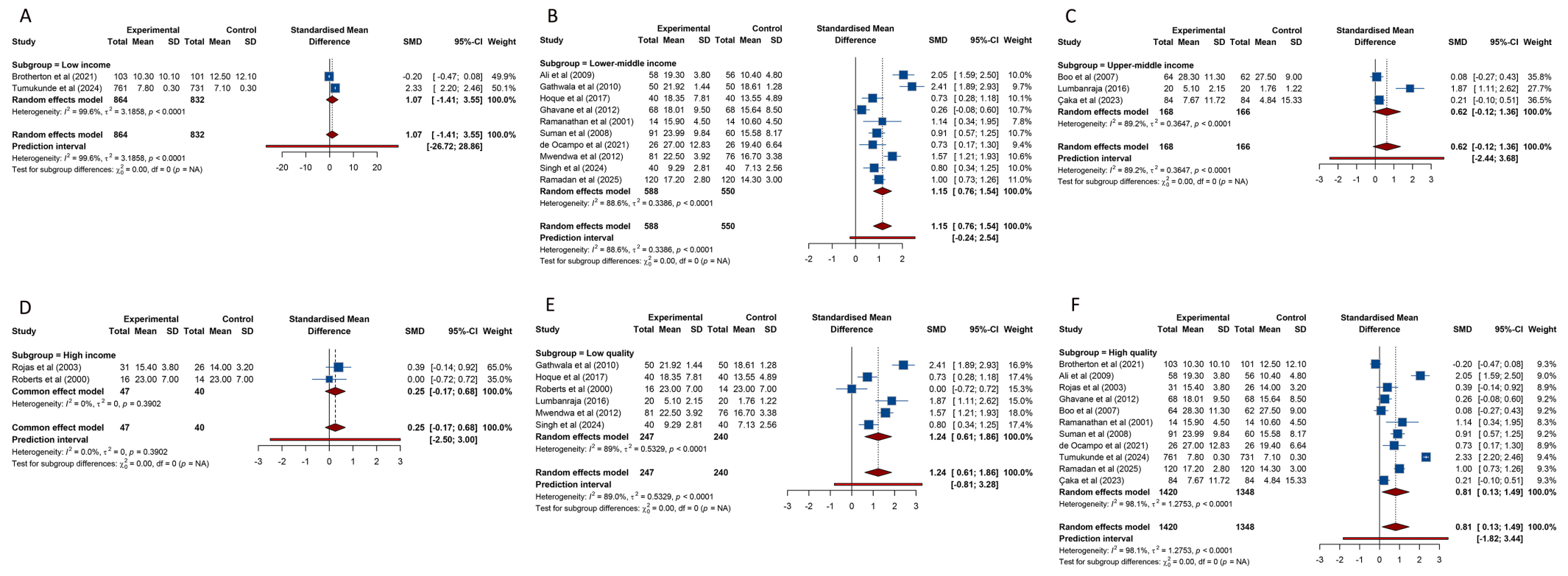


**FIGURE S5** Subgroup analysis of length gain rate. (A) Subgroup = Lower-middle income; (B) Subgroup = High income; (C) Subgroup = Low quality; (D) Subgroup = High quality.


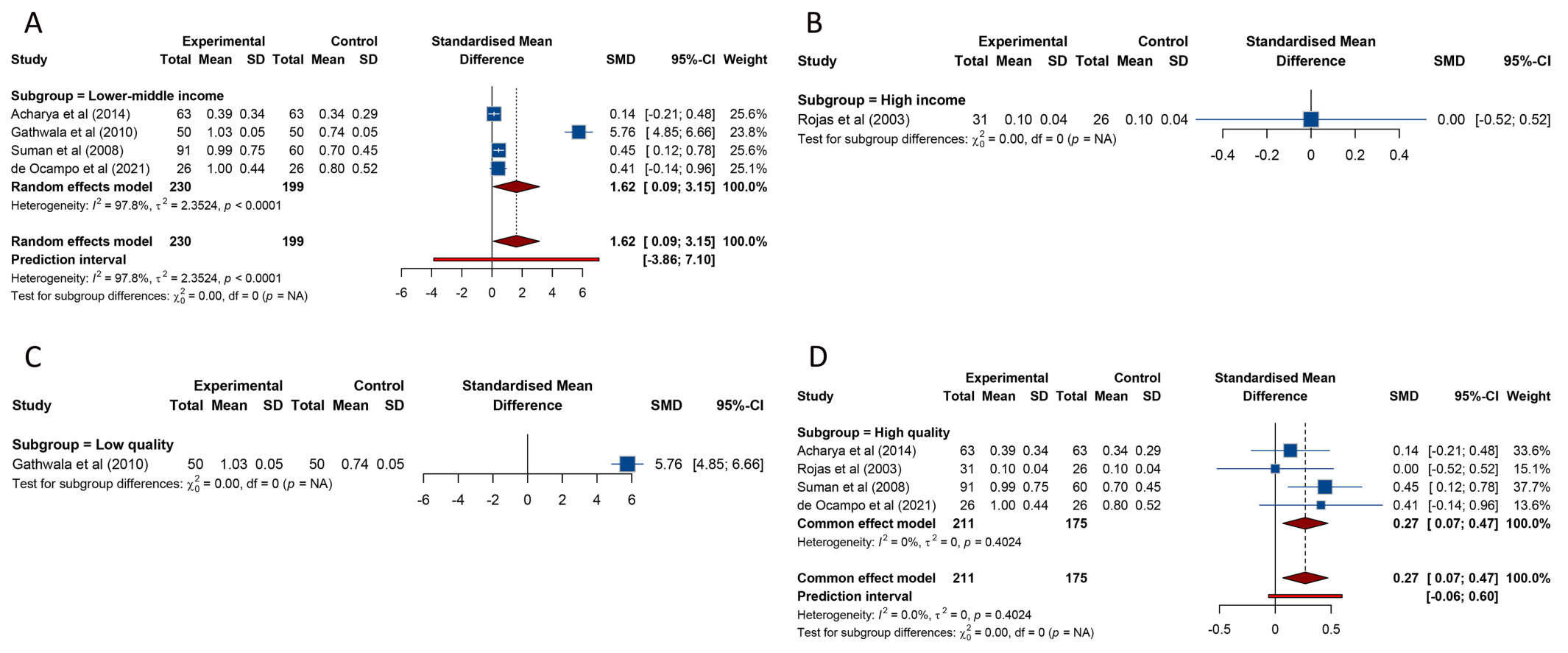


**FIGURE S6** Subgroup analysis for rate of head circumference gain. (A) Subgroup = Lower-middle income; (B) Subgroup = Upper-middle income; (C) Subgroup = High income; (D) Subgroup = Low quality; (E) Subgroup = High quality.


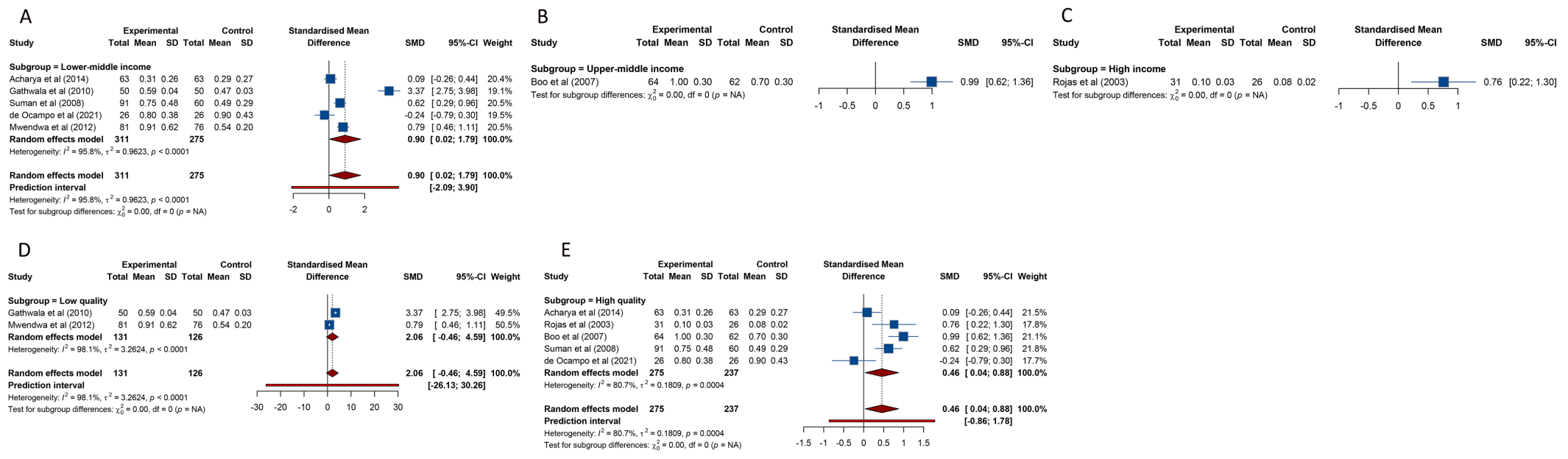


**FIGURE S7** Subgroup analysis of hypothermia. (A) Subgroup = Low income; (B) Subgroup = Lower-middle income; (C) Subgroup = Upper-middle income; (D) Subgroup = High income; (E) Subgroup = Low quality; (F) Subgroup = High quality.


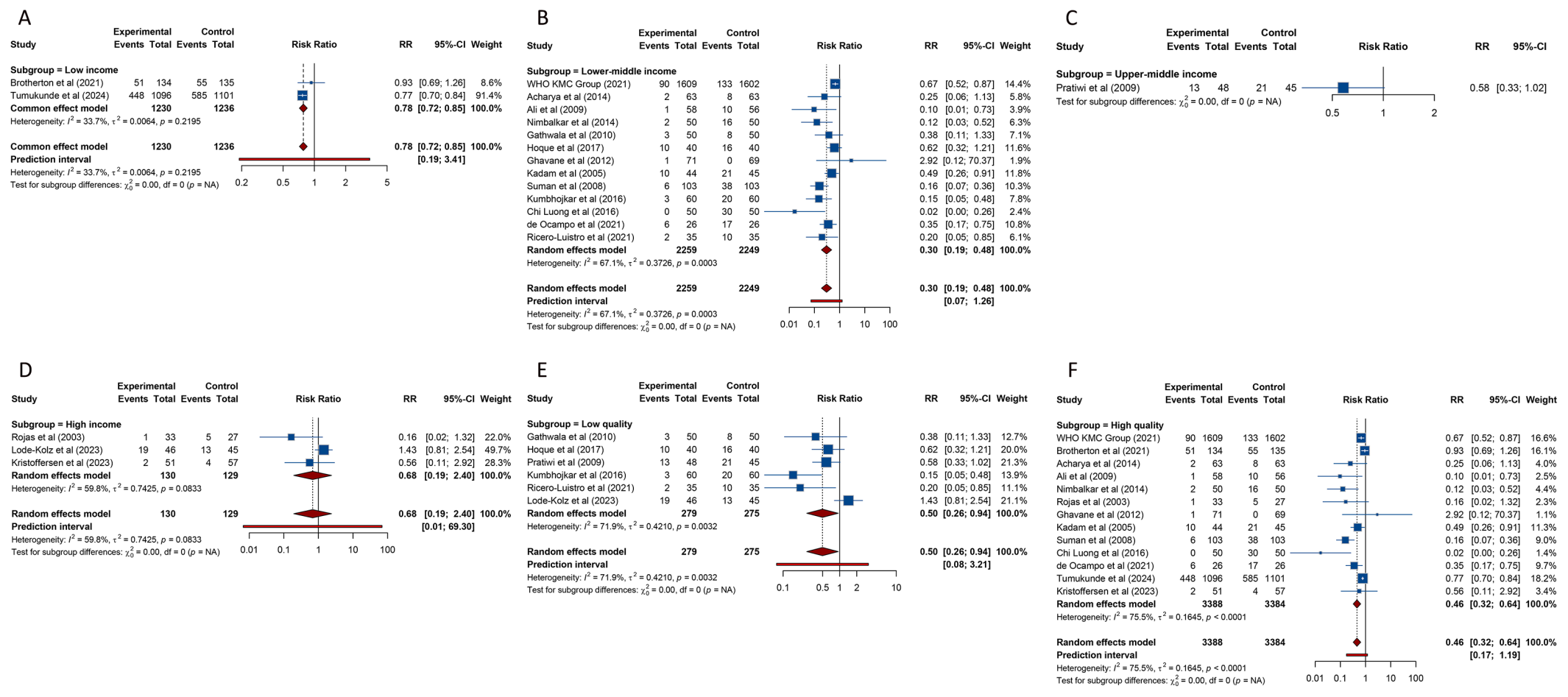


**FIGURE S8** Subgroup analysis of hyperthermia. (A) Subgroup = Low income; (B) Subgroup = Lower-middle income; (C) Subgroup = High income; (D) Subgroup = Low quality; (E) Subgroup = High quality.


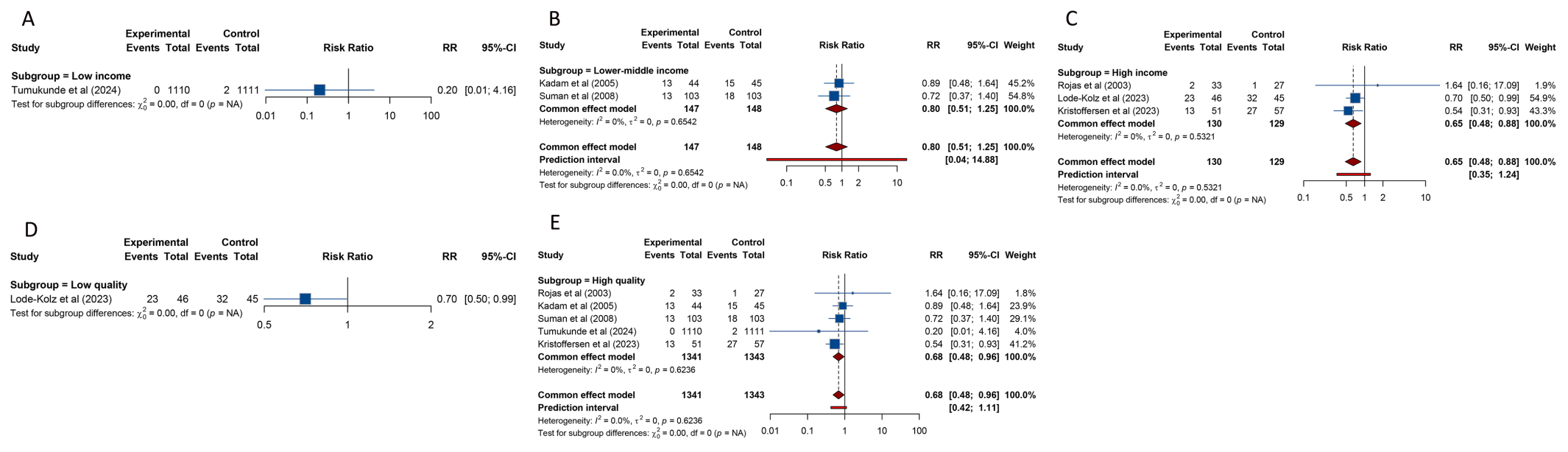


**FIGURE S9** Subgroup analysis of apnea. (A) Subgroup = Low income; (B) Subgroup = Lower-middle income; (C) Subgroup = High income; (D) Subgroup = Low quality; (E) Subgroup = High quality.


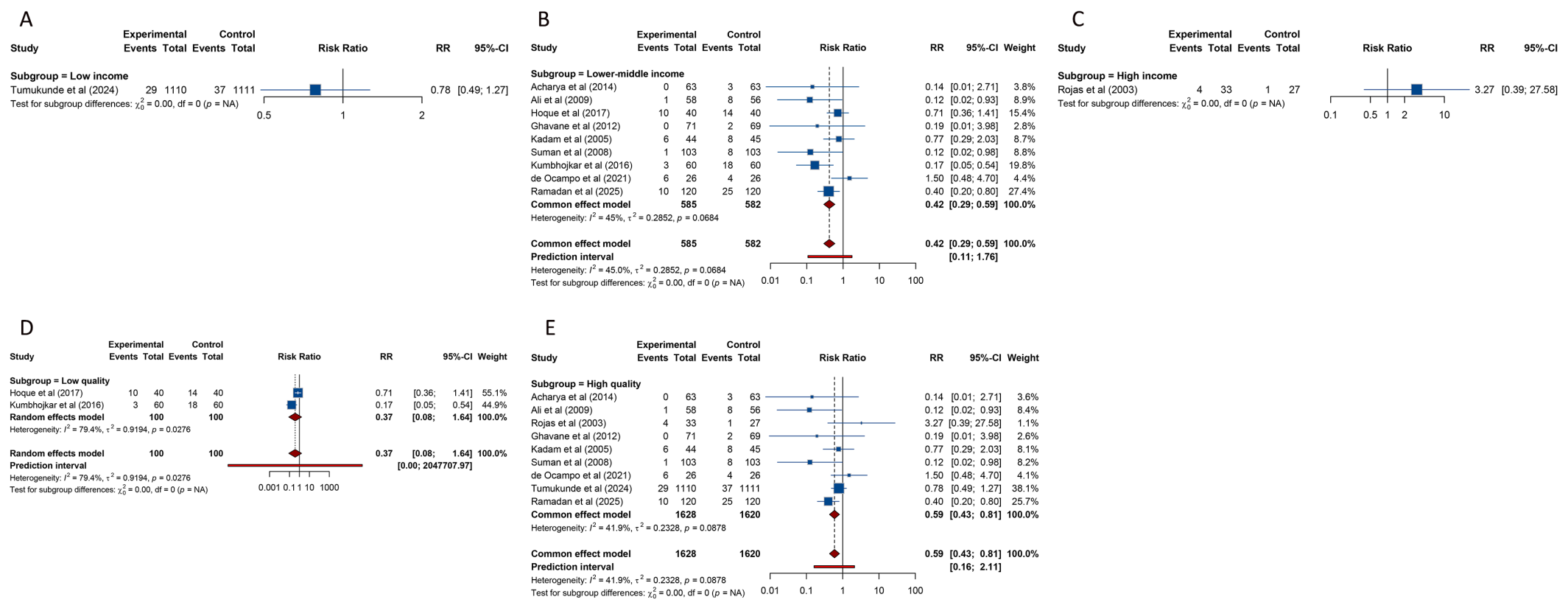


**FIGURE S10** Subgroup analysis of sepsis. (A) Subgroup = Low income; (B) Subgroup = Lower-middle income; (C) Subgroup = Upper-middle income; (D) Subgroup = High income; (E) Subgroup = Low quality; (F) Subgroup = High quality.


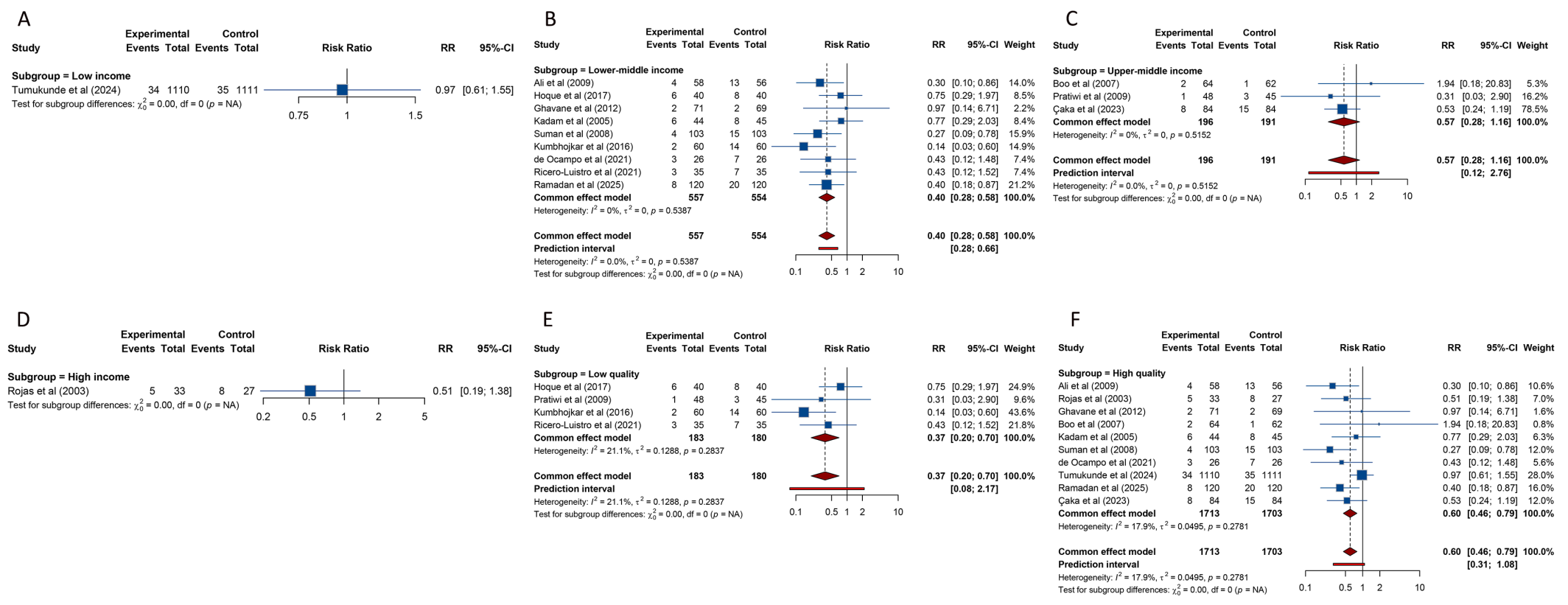


**FIGURE S11** Subgroup analysis of necrotizing enterocolitis. (A) Subgroup = Low income; (B) Subgroup = Lower-middle income; (C) Subgroup = High income; (D) Subgroup = Low quality; (E) Subgroup = High quality.


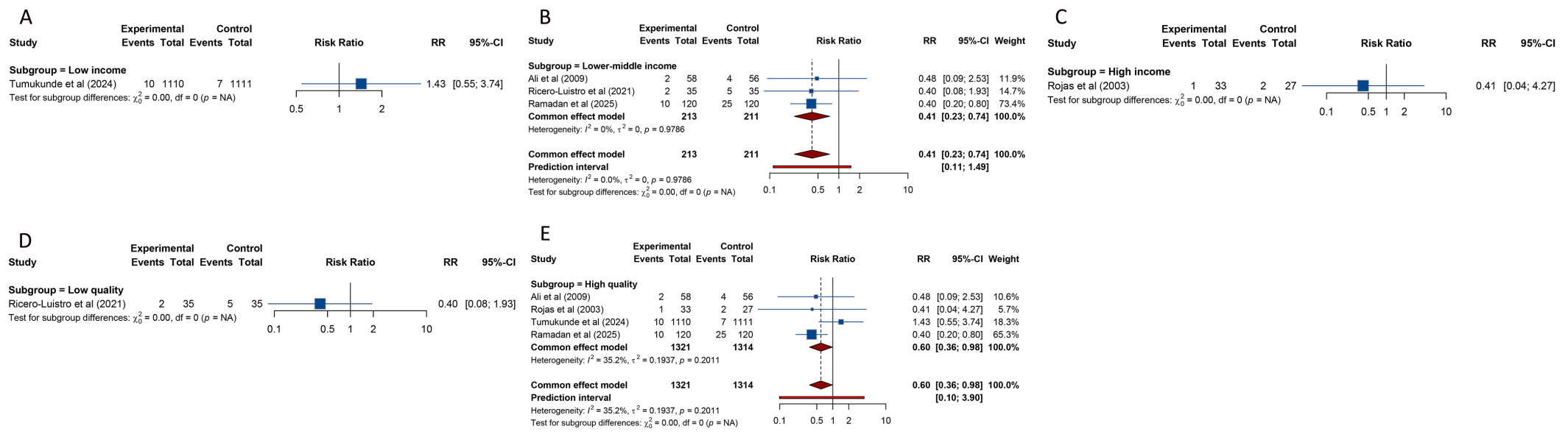


**FIGURE S12** Subgroup analysis of hypoglycemia. (A) Subgroup = Lower-middle income; (B) Subgroup = High income; (C) Subgroup = High quality.


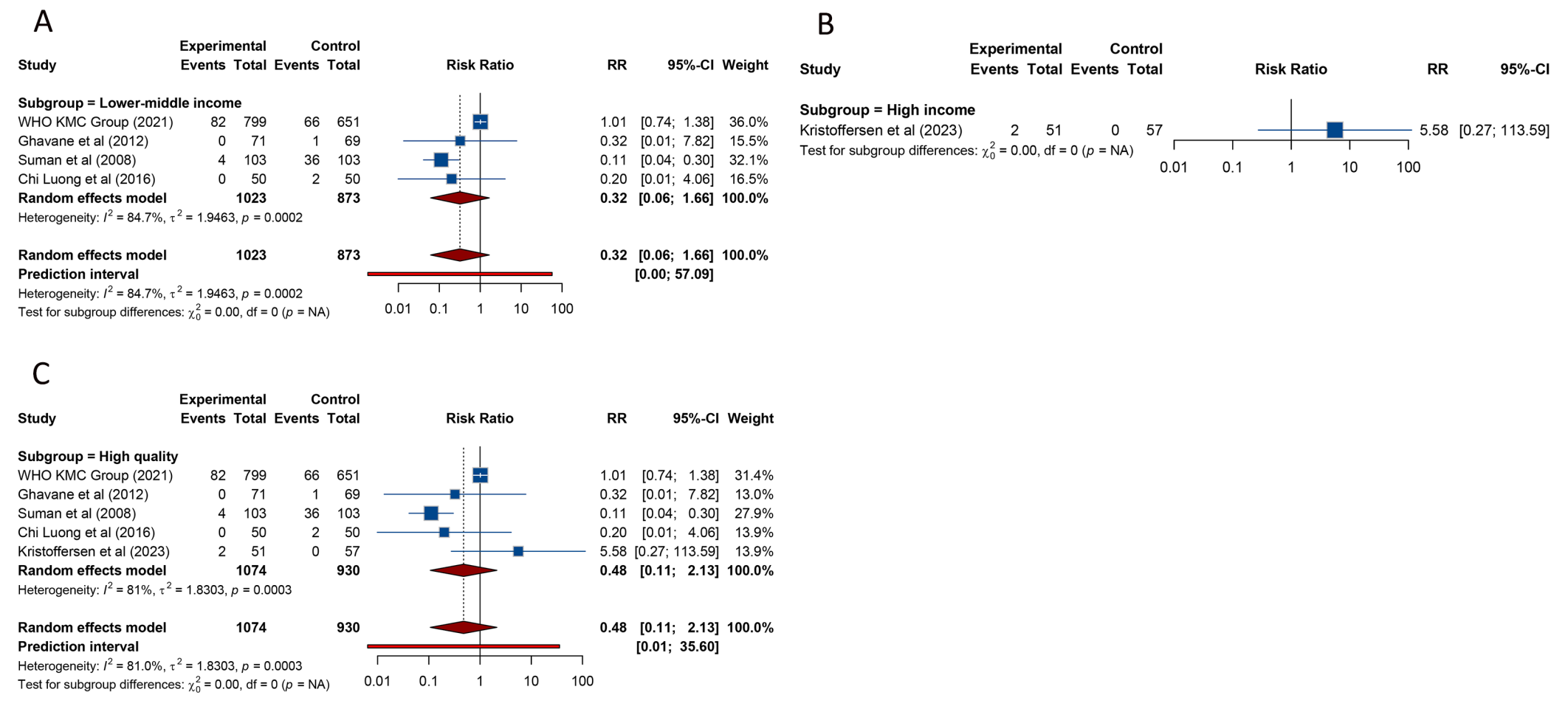


**FIGURE S13** Sensitivity analysis for the outcomes with at least 8 included studies. (A) In-hospital mortality; (B) Length of hospital stay; (C) Weight gain rate; (D) Hypothermia; (E) Apnea; (F) Sepsis.


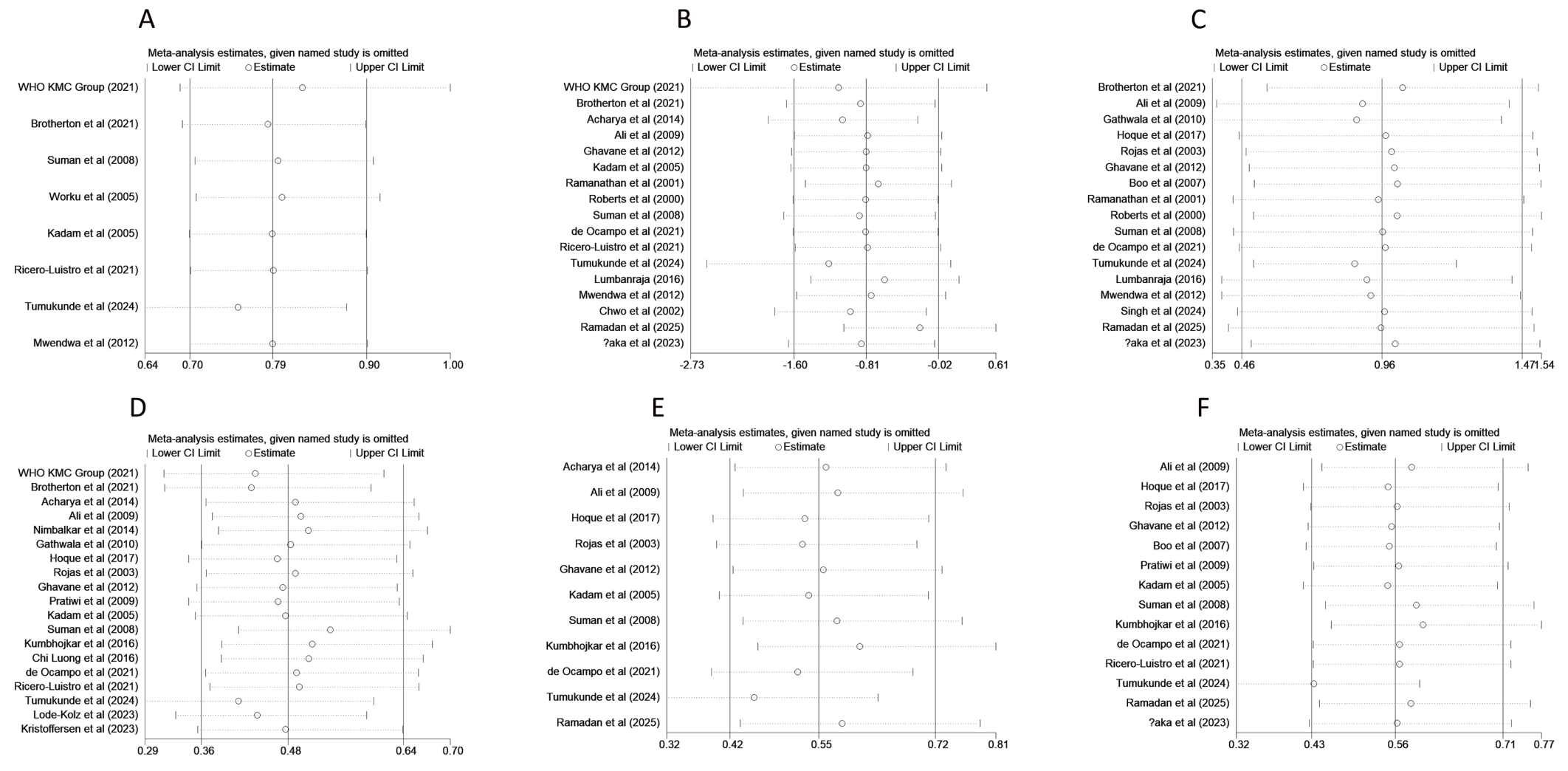


**FIGURE S14** Funnel plots of the outcomes with at least 8 included studies. (A) In-hospital mortality; (B) Length of hospital stay; (C) Weight gain rate; (D) Hypothermia; (E) Apnea; (F) Sepsis.


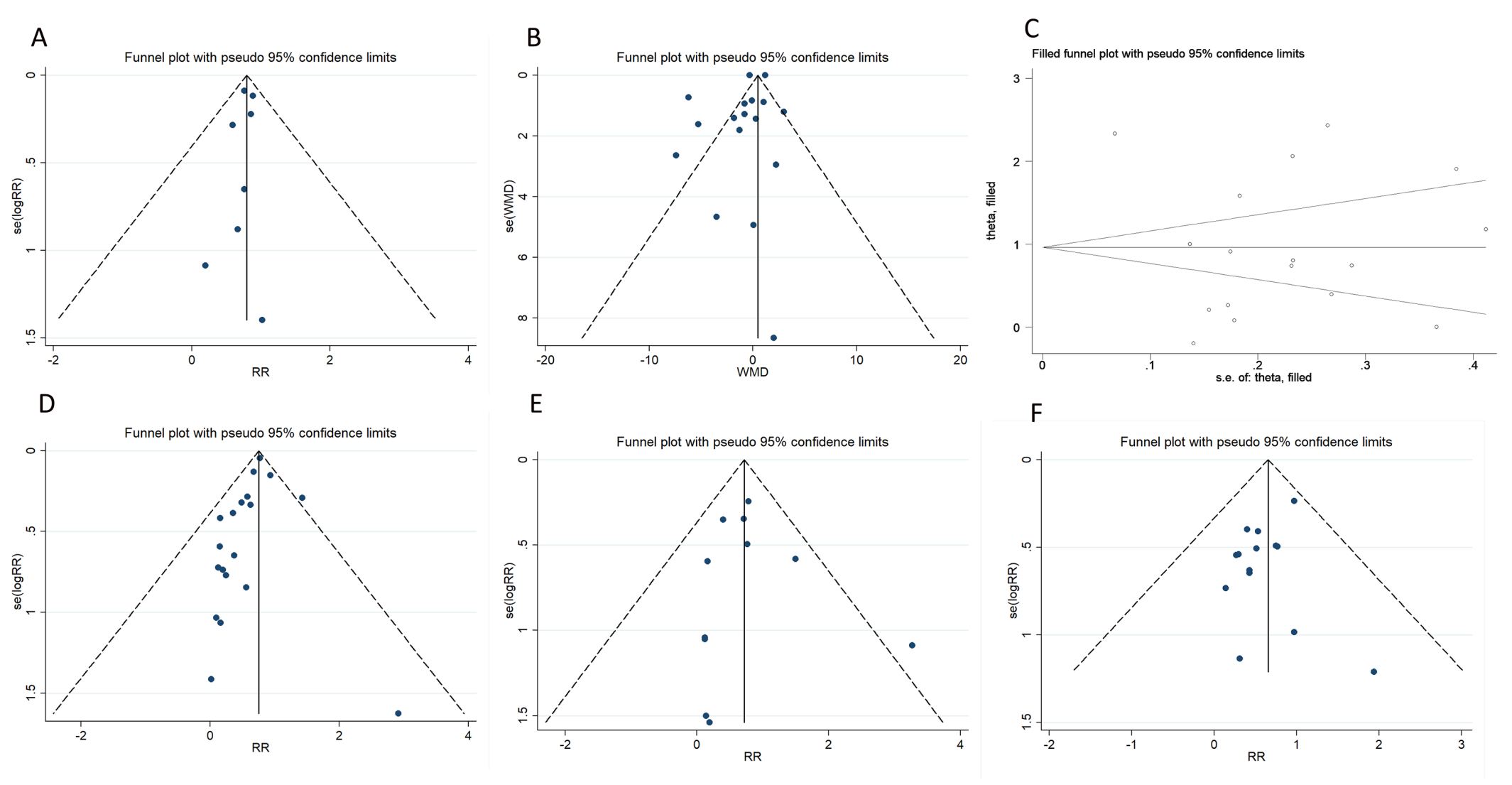

Supplement: Supplementary file 2 [file Data_Sheet_2.docx]
