# Supplementary material for: Efficacy and safety of Kangaroo mother care vs. conventional care during hospitalization for preterm and/or low birth weight infants: a meta-analysis with trial sequential analysis of randomized controlled trials
Source: Front Med (Lausanne). 2026 Jan 14;12:1736973. doi: 10.3389/fmed.2025.1736973 (PMC12847293; doi:10.3389/fmed.2025.1736973)
Supplement: Supplementary file 3 [file Table_1.docx]

| TABLE S1 Quality analysis of the included studies by modified Jadad scale. | | | | | | |
| --- | --- | --- | --- | --- | --- | --- |
| Study | Randomization | Randomization concealment | Double blind | Withdrawals and dropouts | Score | Study quality |
| WHO Immediate KMC Study Group et al (2021) | 2 | 2 | 0 | 1 | 5 | High |
| Brotherton et al (2021) | 2 | 2 | 0 | 1 | 5 | High |
| Acharya et al (2014) | 2 | 1 | 0 | 1 | 4 | High |
| Worku et al (2005) | 2 | 1 | 0 | 0 | 3 | Low |
| Ramadan et al (2025) | 2 | 2 | 0 | 1 | 5 | High |
| Ali et al (2009) | 2 | 1 | 0 | 1 | 4 | High |
| de Ocampo et al (2021) | 2 | 2 | 0 | 0 | 4 | High |
| Nimbalkar et al (2014) | 2 | 2 | 0 | 1 | 5 | High |
| Tumukunde et al (2024) | 2 | 2 | 0 | 1 | 5 | High |
| Gathwala et al (2010) | 2 | 1 | 0 | 0 | 3 | Low |
| Hoque et al (2017) | 1 | 2 | 0 | 0 | 3 | Low |
| Rojas et al (2003) | 2 | 2 | 0 | 1 | 5 | High |
| Ghavane et al (2012) | 2 | 2 | 0 | 1 | 5 | High |
| Lumbanraja (2016) | 1 | 0 | 0 | 1 | 2 | Low |
| Hake-Brooks et al (2008) | 2 | 1 | 0 | 1 | 4 | High |
| Kristoffersen et al (2023) | 2 | 1 | 0 | 1 | 4 | High |
| Boo et al (2007) | 1 | 2 | 0 | 1 | 4 | High |
| Kumbhojkar et al (2016) | 1 | 2 | 0 | 0 | 3 | Low |
| Chwo et al (2002) | 1 | 0 | 0 | 0 | 1 | Low |
| Singh et al (2024) | 1 | 0 | 0 | 0 | 1 | Low |
| Pratiwi et al (2009) | 2 | 0 | 0 | 0 | 2 | Low |
| Mwendwa et al (2012) | 1 | 0 | 0 | 1 | 2 | Low |
| Çaka et al (2023) | 2 | 1 | 0 | 1 | 4 | High |
| Kadam et al (2005) | 1 | 2 | 0 | 1 | 4 | High |
| Chi Luong et al (2016) | 1 | 2 | 0 | 1 | 4 | High |
| Ramanathan et al (2001) | 2 | 1 | 0 | 1 | 4 | High |
| Lode-Kolz et al (2023) | 2 | 1 | 0 | 0 | 3 | Low |
| Roberts et al (2000) | 1 | 2 | 0 | 0 | 3 | Low |
| Ricero-Luistro et al (2021) | 1 | 2 | 0 | 0 | 3 | Low |
| Suman et al (2008) | 1 | 2 | 0 | 1 | 4 | High |
| Manzoor et al (2023) | 2 | 0 | 0 | 0 | 2 | Low |
